# A comprehensive review on graphene-/graphitic carbon nitride-based MOFs for the photocatalytic purification of dye wastewater

**DOI:** 10.1039/d6ra01722c

**Published:** 2026-05-11

**Authors:** Stephen Sunday Emmanuel, Gloria Onome Achurefe, Sefiu Olaitan Amusat, Ademidun Adeola Adesibikan, Ehiaghe Agbovhimen Elimian, Simiso Dube, Mathew Muzi, Mustapha Omenesa Idris

**Affiliations:** a Department of Industrial Chemistry, Faculty of Physical Sciences, University of Ilorin P. M. B. 1515 Ilorin Nigeria stephenemmanuel6011@gmail.com; b Department of Chemical Engineering, University of Lagos Akoka, Yaba Lagos Nigeria; c Department of Chemistry, College of Science, Engineering and Technology, University of South Africa Florida Campus, Christian De Wet & Pioneer Avenue Florida 1709 South Africa; d Department of Civil and Environmental Engineering, University of Alberta T6G1H9 Edmonton AB Canada; e Interdisciplinary Research Center for Membranes and Water Security, King Fahd University of Petroleum and Minerals Dhahran 31261 Saudi Arabia

## Abstract

The development of advanced porous functional materials with enhanced photocatalytic performance for the degradation of hazardous pollutants such as dyes has led to the emergence of graphene-/graphitic carbon nitride-based metal–organic frameworks (GBMOFs) as promising photocatalysts. This study aims to comprehensively review advances in GBMOFs for the photocatalytic degradation of dye pollutants. As part of the study objective, the photocatalytic performance, recyclability, and stability dynamics of GBMOFs were critically reviewed to identify patterns and key trends. Emerging trends and technology readiness levels were succinctly discussed to assess the industrial applicability of GBMOF photocatalytic systems for dye remediation. Interestingly, the findings revealed that most GBMOF systems can deliver >80% degradation efficiency at pH 3–13 and doses of 2–1000 mg in 3–390 minutes. A predominant operational trend involving ˙OH and ˙O_2_^−^ was also noticed. Furthermore, the study shows that GBMOFs can be recycled 3–30 times while maintaining >70% of their original degradation performance and morphology in most of the cases. Recent advances and emerging trends include the application of p–n–p heterojunctions, ternary GBMOFs (which outperformed binary counterparts), piezo and sono-assisted photocatalysis, the use of natural sunlight, oxygen vacancies, and defect engineering. Finally, future perspectives are presented to establish a clear framework for guiding the future design of highly efficient, stable, practical, and enhanced GBMOF photocatalytic systems for the remediation of various dyes and pollutants.

## Abstract 2

The development of advanced porous functional materials with enhanced photocatalytic performance for the degradation of hazardous dye pollutants has led to the emergence of graphene/graphitic carbon nitride-based MOFs as promising photocatalysts.

## Graphical abstract

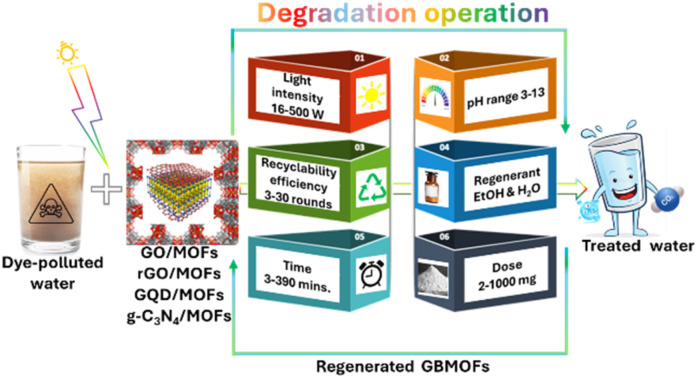

## 1. Introduction

Water pollution caused by synthetic dyes has become a global environmental concern due to the continuous discharge of dye-containing effluents into ecosystems.^1^ A significant proportion of this pollution originates from untreated or poorly treated wastewater released from industries such as textiles, printing, pharmaceuticals, food, paper, and leather.^2^ The continuous and widespread use of synthetic dyes in these aforementioned industries has further intensified their uncontrolled release into aquatic systems.^3^ Unfortunately, the accumulation of these dyes poses serious environmental and health risks, including toxicity, allergic reactions, and ecological damage, particularly in developing regions where wastewater treatment infrastructure remains inadequate.^4–8^ Consequently, the development of sustainable and effective dye remediation technologies has become increasingly urgent.

Conventional treatment methods, such as adsorption, coagulation, and biological degradation, have been widely investigated for dye removal.^9–11^ However, these approaches suffer from inherent limitations, including incomplete degradation, the generation of secondary pollutants, and reduced degradation efficiency for stable or soluble dyes.^9–11^ As a result, these methods are often insufficient for complete mineralization of dye contaminants.

Photocatalysis has emerged as a promising green technology for wastewater treatment, operating under ambient conditions and utilizing solar energy.^12–16^ As an advanced oxidation process (AOP), photocatalysis employs semiconductor materials such as TiO_2_, ZnO, and g-C_3_N_4_ to generate reactive oxygen species (*e.g.*, ˙OH radicals) under light irradiation, leading to the mineralization of organic pollutants into CO_2_ and H_2_O.^12–16^ Despite its advantages, photocatalysis is limited by challenges such as rapid electron–hole (e^−^/h^+^) recombination, photo-corrosion, and poor visible-light absorption in some materials,^17–19^ necessitating the development of more efficient photocatalysts.

Metal–organic frameworks (MOFs), a class of porous crystalline materials composed of metal ions coordinated with organic ligands, have gained significant attention for photocatalytic applications due to their high surface area, tunable structures, and abundant active sites.^20,21^ Various MOFs have been explored for dye degradation,^22–26^ with their performance greatly influenced by the nature of metal clusters.^27^ For instance, Fe-based MOFs, such as MIL-53(Fe), have demonstrated enhanced photocatalytic activity, particularly when modified with oxidants like H_2_O_2_.^28,29^ Similarly, Zr-based MOFs (*e.g.*, UiO-66) and Ti-based MOFs (*e.g.*, NTU-9) have shown promising photocatalytic performance for degrading dyes such as methyl orange, methylene blue, and rhodamine B.^30–35^ However, MOFs also exhibit limitations, including a limited light-absorption range, low charge mobility, and structural instability, under certain conditions.^36^

To address these challenges, the integration of MOFs with functional materials has been widely explored. In particular, graphene and its derivatives have attracted attention due to their exceptional electrical conductivity, thermal stability, and large surface area.^37–42^ Among these, graphitic carbon nitride (g-C_3_N_4_) stands out as a metal-free, visible-light-active photocatalyst with a suitable band gap.^43,44^ The combination of MOFs with graphene and g-C_3_N_4_-based materials enables synergistic interactions that enhance charge separation, improve light absorption, and increase overall photocatalytic efficiency.^37,38,45^ These distinctive synergetic properties of graphene-/graphitic carbon nitride-based materials, combined with those of MOFs, make them promising candidates for engineering functional composite materials such as GBMOFs.^45^ Consequently, quite a lot of research on the photocatalytic degradation of dye pollutants using GBMOFs has been carried out, and, within the authors' extensive literature survey, few reviews are available in this direction. For example, Qu,^46^ Naser,^47^ and Kumari's^48^ team reviewed the preparation, characteristics, and general applications of GO/MOFs. Another research group^49^ reviewed g-C_3_N_4_/MOFs for photocatalytic water splitting and hydrogen evolution. Recently, a review work was written on graphene/g-C_3_N_4_-based MOFs for the degradation of pharmaceutical pollutants with emphasis on efficiency and reusability.^50^ The focus and significance of these existing reviews are summarized in Table 1. In comparison, the present work takes its novelty in being the first standalone review to critically evaluate the performance and technology readiness level of graphene-/graphitic carbon nitride-based MOFs directly for the photocatalytic degradation of all types of dye pollutants. The main goal of this review is to comprehensively evaluate and integrate the recent advancements in GBMOF composites towards the photocatalytic degradation of organic dye contaminants. This review aims to clarify the impact of the synergistic interaction of the functionalized composites towards overcoming the inherent photocatalytic setbacks of each of the respective GBMOF components. By including a thorough systematic discussion of degradation efficiencies and limitations, this review seeks to establish a clear framework for guiding the design of highly efficient, stable, and practical photocatalytic systems for various organic dyes using GBMOFs. As part of the study objective, the recoverability, recyclability performance, and stability dynamics of GBMOFs are critically discussed to identify patterns and present recent advances. Furthermore, key trends, metal leaching potential, and perspectives on technology readiness level are succinctly discussed to assess the industrial applicability of GBMOF photocatalytic systems for dye remediation. Finally, future research hotspots and prospects are presented in order to spark innovative thoughts in future researchers.

**Table 1:** Comparison of the present work's focus with existing related reviews

Year	The primary focus of the review	References (author)
2021	Preparation, characteristics, and general applications of GO/MOFs	46
2023	Synthesis, properties, and applications of MOFs supported on GO	47
2022	Synthesis and applications of MOFs and graphene-based composites	48
2022	g-C_3_N_4_/MOFs for photocatalytic water splitting and hydrogen evolution	49
2025	Graphene/g-C_3_N_4_-based MOFs for the degradation of pharmaceutical pollutants	50
2025	MOF composites functionalized with metallic NPs, quantum dots, oxides, and zeolites for the detection, adsorption, and degradation of heavy metals, dyes, pesticides, and pharmaceuticals	51
2021	MOF/polymer composites for the photodegradation of organic dyes	52
2024	MXene/MOF composites for pollutant photocatalytic degradation with special emphasis on synthesis and efficiency	53
2020	Synthesis and application of MOF@quantum dots for CO_2_ reduction, H_2_ production, organic dye degradation, NO oxidation and Cr(vi) reduction	54
2023	MOF@cellulose for the remediation of metals, oils, dyes, pharmaceuticals, and pesticides through adsorption, photocatalysis, and membrane separation techniques	55
2026	Graphene-/graphitic carbon nitride-based metal–organic frameworks for the photocatalytic degradation of dye pollutants with special focus on recent advances, photocatalytic performance, recoverability, recyclability efficiency, stability dynamics, metal leaching potential, emerging trends, and technology readiness level	This review

## 2. Review methodology

**Fig. 1:**
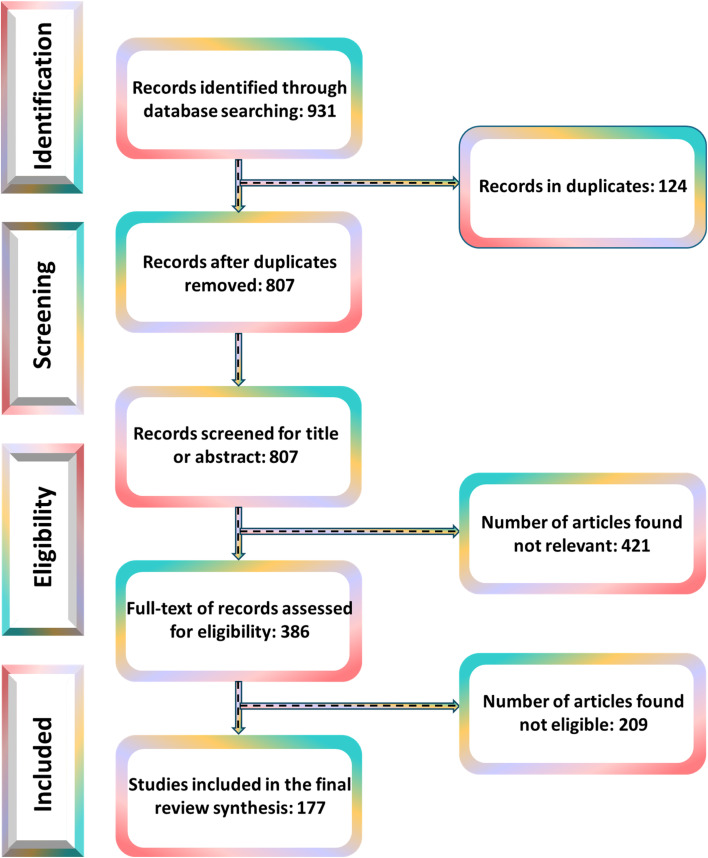
Schematic overview of the PRISMA flow diagram.^56^ Adapted from Bayode *et al.*,^56^ 2024, licensed under CC BY.

A systematic literature search was conducted following the PRISMA guidelines (Preferred Reporting Items for Systematic Reviews and Meta-Analyses). The search was performed across multiple reputable scientific databases, including Scopus, Google Scholar, ScienceDirect, and PubMed, covering all relevant publications from 2014 up to April 2026. The literature search focused on the topic of advanced functional materials (particularly MOFs, graphene, GO, rGO, and g-C_3_N_4_) for photocatalytic degradation of hazardous pollutants, with special emphasis on dye degradation. Key search terms included: metal–organic frameworks (MOFs), graphene-based MOFs, graphitic carbon nitride-based MOFs, graphitic carbon nitride (g-C_3_N_4_), graphene, photocatalysis, dye degradation, and dye wastewater treatment. Boolean search strings, including g-C_3_N_4_, graphene oxide, reduced graphene oxide, and dye removal, were also employed. Additional terms, such as technology readiness level, were used to capture recent advances and practical applicability. These keywords were used in various combinations using Boolean operators (“AND” and “OR”) to optimize the retrieval of relevant articles. After removing duplicates, the retrieved articles were screened based on predefined inclusion and exclusion criteria. Only peer-reviewed articles published in English were included. Articles such as opinions, corrigenda, retracted papers, conference abstracts, encyclopedias, and seminars were excluded. Additionally, papers where the full text was not available were not considered. The screening and selection process was performed collaboratively by all authors. A total of 177 articles were ultimately used in the preparation of this review. A schematic overview of the PRISMA flow diagram is presented in Fig. 1.

## 3. Functional advantages of GBMOFs

**Fig. 2:**
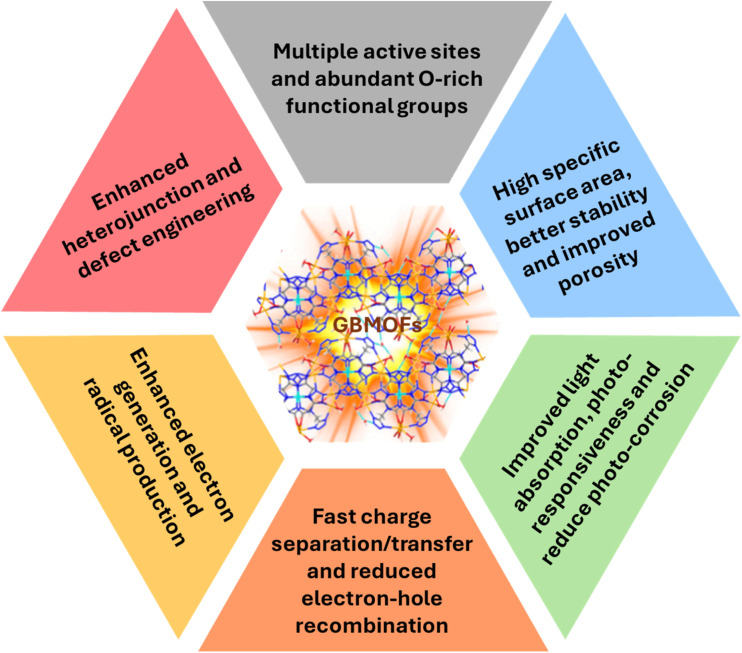
Unique functional advantages of graphene-/graphitic carbon nitride-based metal–organic frameworks within the context of photocatalysis.

As presented in Fig. 2, GBMOFs exhibit unique functional advantages arising from the synergistic coupling of highly conductive and photosensitive carbon networks with porous crystalline frameworks. Firstly, the incorporation of graphene/GO/rGO significantly enhances charge transport and electron mobility, allowing rapid extraction and shuttling of photogenerated electrons from MOF active sites and efficiently reducing e^−^/h^+^ recombination. Simultaneously, the intercalation of MOF crystals between the graphene sheets stops the two-dimensional layers from restacking, a typical problem that affects pure graphene-based materials and reduces their surface area and performance in catalysis and supercapacitors.^50,57^ On the other hand, g-C_3_N_4_ materials improve light utilization by acting as photosensitizers and broadening absorption into the visible region, while their π-conjugated structures promote strong π–π interactions with aromatic dye pollutant molecules, increasing adsorption efficiency.^50,58,59^ Moreover, graphene-/g-C_3_N_4_-based materials exhibit a large interfacial contact area and reduced photo-corrosion and leaching and provide abundant anchoring sites, enabling uniform MOF growth and improved structural stability while preventing particle agglomeration. Also, graphene-based materials such as GO/rGO can enhance the coordination bonding between metal nodes and organic ligands in MOFs, due to their unique sp^2^ domains.^50,60^ Additionally, graphene often functions as an electron tank and mediator, boosting stability in humid environments and facilitating directional charge transfer in GBMOF complex heterojunction systems.^50,61^ These combined advantages lead to enhanced photocatalytic activity, as discussed in the next section.

## 4. Synthesis of GBMOFs

**Fig. 3:**
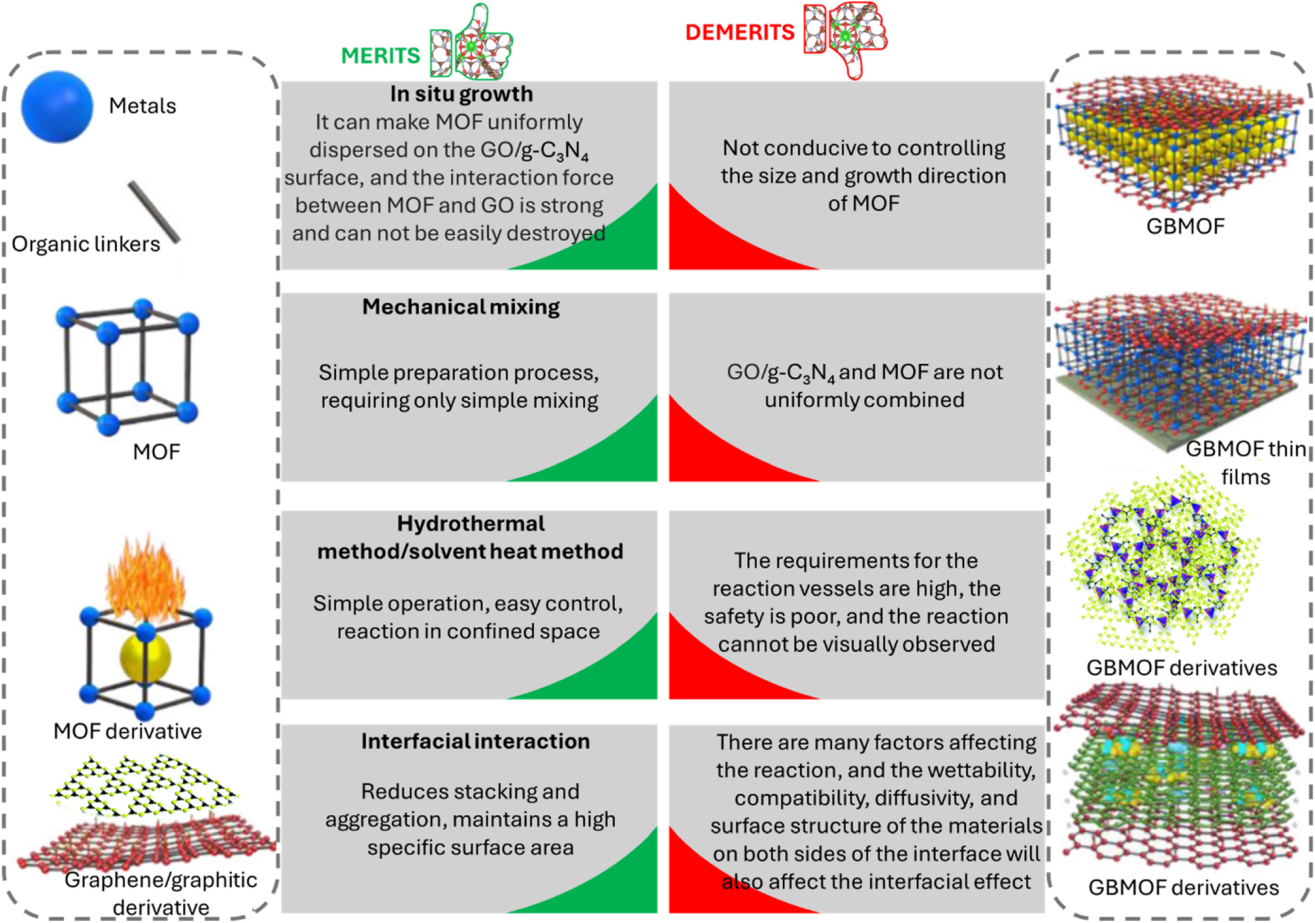
Advantages/disadvantages of selected common methods of GBMOF synthesis.^46,64,65^

Several methods for the synthesis of GBMOFs are available, and a few methodologies for improving their performance have been presented in the literature.^48^ Some of these synthesis strategies involve physical blending of MOFs and graphene/g-C_3_N_4_-based materials, *in situ* growth of MOFs on graphene/g-C_3_N_4_-based materials, and hydrothermal/solvent heat synthesis.^48,62^ All these synthesis procedures and the morphological properties of the resulting GBMOFs are generously discussed in previous studies.^46,63^ The advantages and disadvantages of these methods are concisely presented in Fig. 3.

## 5. Fundamental GBMOF photocatalytic degradation mechanism

Generally, the enhanced photocatalytic performance of GBMOFs arises not merely from conventional heterojunction formation, but from synergistic electronic interactions between the MOF, a semiconductor component g-C_3_N_4_, and graphene derivatives (GO/rGO).^61,66,67^ These interactions collectively regulate light absorption, charge-carrier behaviour, and interfacial redox reactions. Upon light irradiation (*hν* ≥ *E*_g_), photoexcitation generates e^−^/h^+^; however, the subsequent charge-carrier migration pathways are strongly dictated by system-specific band alignment and the presence of graphene as a conductive mediator, rather than by classical semiconductor behavior alone.^61,67^

**Fig. 4:**
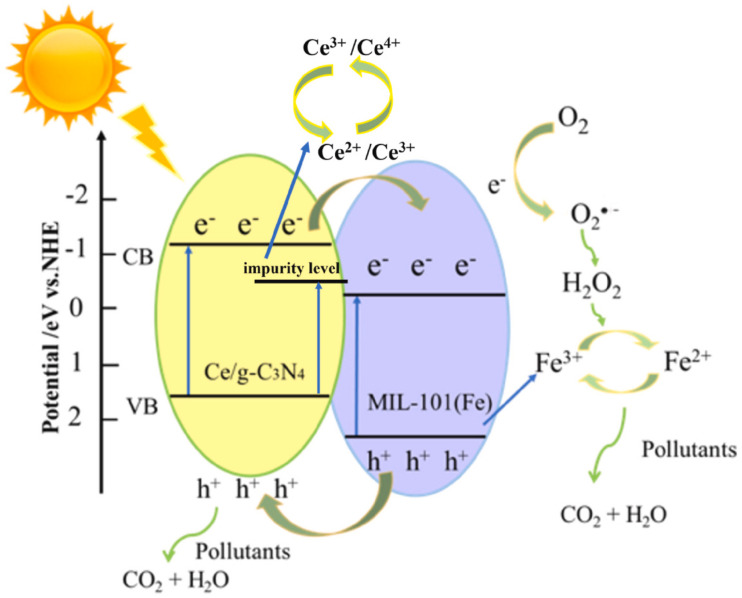
Diagram of type II heterojunction dye degradation mechanism in the MIL-101(Fe)/Ce/g-C_3_N_4_ system under light illumination. Reproduced from ref. 69 with permission from Elsevier B.V., X. Zhang, Z. Song, X. Yu, X. Dong, Y. Peng, K. Wei, L. Cao, X. He, Z. Zhang and J. Fan, *J. Solid State Chem.*, 2023, **323**, 124013, copyright 2023.

Importantly, the relative band positions in GBMOFs are not universal and cannot just be assumed. For example, according to various band gap analyses, the conduction band (CB) of g-C_3_N_4_ (typically −1.1 to −1.3 V *vs.* NHE) is more negative than that of many Zr-based MOFs (*e.g.*, UiO-type frameworks, ≈−0.5 to −0.8 V *vs.* NHE).^68–70^ This alignment thermodynamically favors electron transfer from the g-C_3_N_4_ CB to the MOF CB through the inner electric field generated in a conventional Type-II heterojunction, as shown in Fig. 4.^70,71^ So, the generated electrons gathered in the CB of the MOF will migrate to the CB position of the g-C_3_N_4_ as an electron tank, and the electrons in the CB of the MOF will react with H_2_O_2_ to form ˙OH, as shown in the case of MIL-101(Fe)/Ce/g-C_3_N_4_ and UiO-66/g-C_3_N_4_ for MB and RhB degradation (Fig. 4).^69,71,72^ Notably, because it is the MOF that transports the electron generated by the g-C_3_N_4_, the recombination of charge carriers is reduced.^70^ While the VB of MOF is more positive than that of g-C_3_N_4_, the h^+^ in the VB of MOF will be relocated to the VB of g-C_3_N_4_, so the generated e^−^/h^+^ are effectively separated in space.^69^ In a conflicting mechanism elucidation, Zhang *et al.*^73^ opined that photo-generated electrons of UiO-66 reduce molecular O_2_ to form ˙O_2_^−^ as against the usual norm of reacting with H_2_O_2_ to form ˙OH.

**Fig. 5:**
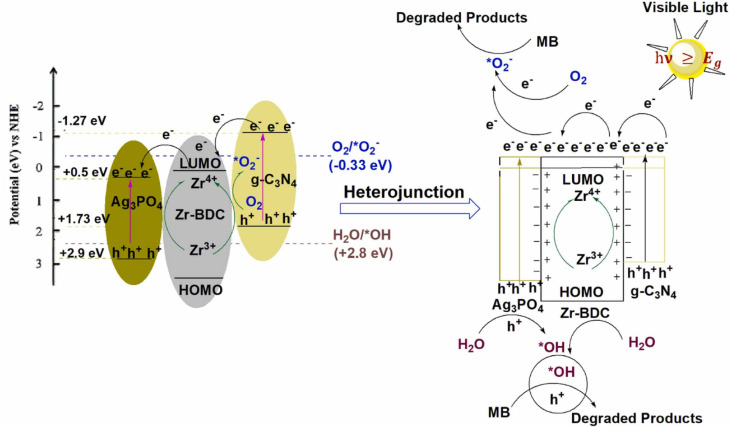
Mechanism of MB degradation using g-C_3_N_4_/UiO-66(Zr)/Ag_3_PO_4_. Reproduced from ref. 75 with permission from Elsevier B.V., J. M. Yassin, A. M. Taddesse and M. Sánchez-Sánchez, *Catal. Today*, 2022, **390–391**, 162–175, copyright 2021.

However, the band alignment is highly sensitive to the MOF's metal node (Zr, Fe, Ti, Cu), oxo-clusters of MOF inter-valence electron transfer, and linker functionalization.^73,74^ This is because the e^−^ in the CB of graphene/g-C_3_N_4_ can move to the LUMO of the MOF linker in GBMOF, and the e^−^ directly migrates to the metal node oxo cluster *via* ligand-to-metal charge transfer, causing reduction of this metal node.^75,76^ This is exemplified in the photocatalytic degradation of MB using g-C_3_N_4_/UiO-66(Zr)/Ag_3_PO_4_, as shown in Fig. 5.^75^ A key limitation here is that the heterojunction type is not explicitly clarified.

**Fig. 6:**
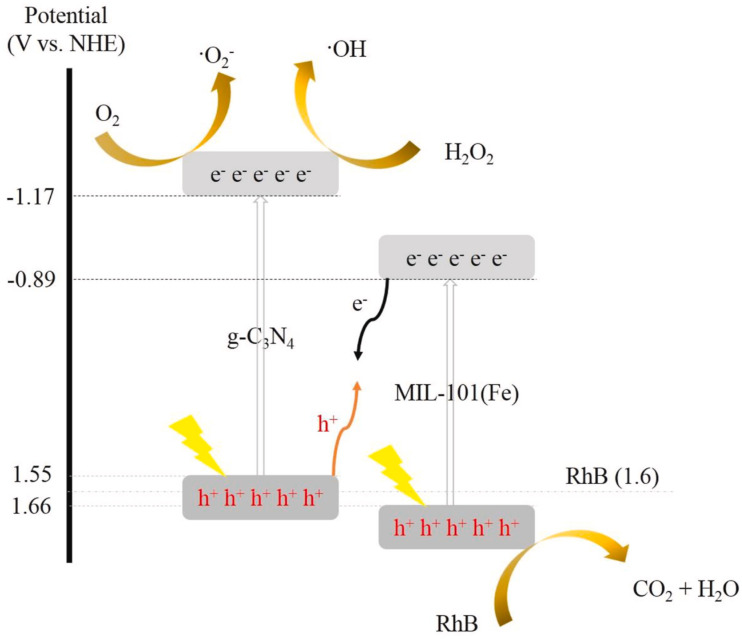
Schematic of the Z-scheme mechanism of MIL-101(Fe)@g-C_3_N_4_ for RhB photodegradation. Reproduced from ref. 77 with permission from Elsevier B.V., R. Kamandi, N. M. Mahmoodi and M. Kazemeini, *Mater. Chem. Phys.*, 2021, **269**, 124726, copyright 2021.

Interestingly, in Fe-based MOFs, such as MIL-101(Fe) and MIL-88A GBMOFs, the CB is often positioned such that a direct Z-scheme mechanism dominates when coupled with GO or g-C_3_N_4_, with photogenerated electrons in the MOF CB recombining with holes in the GO/g-C_3_N_4_ VB, as shown in Fig. 6.^77–79^ Generally, both the CB and VB of MOFs lie below those of GO/g-C_3_N_4_, causing the charge-carrier transport to proceed through a Z-scheme pathway.^77,79^ However, this is not true in all cases, as demonstrated by Tang *et al.*^76^ for the Z-scheme MIL-125(Ti)@GO. Thus, assuming a fixed band alignment/arrangement across all GBMOFs leads to misleading mechanistic interpretations. Moreover, in the case of GBMOF specially doped with metallic NP such as Ce, the unfilled electron orbitals can trap the photogenerated electrons and therefore restrain the charge carriers' recombination or redirect their pathways.^69^

In contrast to traditional descriptions, GO and rGO do not function as photosensitizers that inject electrons into the MOF. Owing to their high electrical conductivity and favorable Fermi level (≈−4.5 to −4.8 eV), GO/rGO acts exclusively as electron acceptors/trappers, support, and conductive pathways.^80–82^ Photogenerated electrons from either the semiconductor or MOF component are preferentially transferred to the graphene/g-C_3_N_4_ sheets, where they are rapidly delocalized, and this charge carrier pathway prolongs e^−^/h^+^ lifetime.^72,80^ In the case of ternary GBMOFs, GO/rGO transport charge carriers from the semiconductor (*e.g.*, Ag_3_VO_4_) to the MOF.^81^ However, Tang *et al.*^76^ reported a conflicting mechanism, which states that in Z-scheme GO@MIL-125(Ti), GO acts as the e^−^ donor and MOF acts as the electron acceptor, thus, e^−^ in the GO CB moved to the VB of MIL-125(Ti). On a general note, direct electron transfer from GO/rGO to the MOF is thermodynamically unfavorable and hardly proceeds because the Fermi level of graphene lies below the CB of common MOFs.^80^ This electron-trapping role suppresses e^−^/h^+^ recombination and creates efficient interfacial charge-transport channels or alternative charge-transfer pathways, such as Z- or S-scheme mechanisms.^66^

**Fig. 7:**
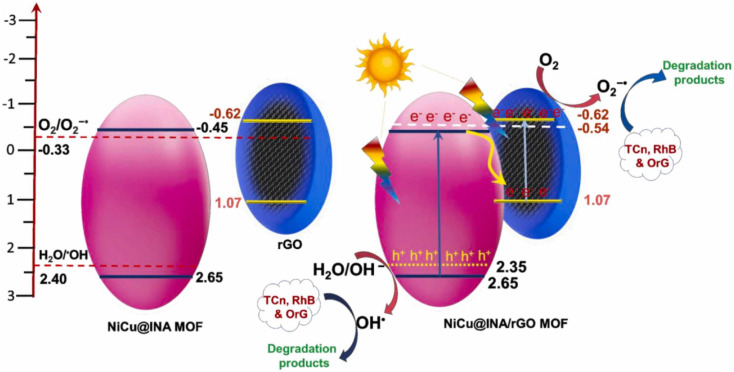
Schematic of the Z-scheme mechanism of rGO@NiCu-MOF for OrG photodegradation. Reproduced from ref. 83 with permission from Elsevier B.V., K. Divyarani, S. Sreenivasa, V. S. Anusuaya devi, M. S. Raghu, T. M. C. Rao, F. Alharethy, B. H. Jeon, P. Martis, S. Kumar and L. Parashuram, *Colloids Surf., A*, 2024, **703**, 135415, copyright 2024.

In many GBMOF photocatalytic systems, Z-scheme or S-scheme charge-carrier pathways are more beneficial. Specifically, in a Z-scheme configuration, electrons in the CB of one component recombine with holes in the VB of the other *via* the graphene mediator, preserving highly reductive electrons and strongly oxidative holes (usually on the MOF).^83,84^ This was demonstrated in the degradation of OrG by rGO@NiCu-MOF, as shown in Fig. 7.^83^ In contrast, in the case of the Ni-MOF/g-C_3_N_4_ Z-scheme, electrons in the MOF CB recombine with the h^+^ in the VB of g-C_3_N_4_.^85^ One critical gray area noted here lies in the system-dependent and sometimes reversed charge carrier transfer pathways in Z-scheme systems, where the direction of e^−^/h^+^ recombination varies with material composition, making it difficult to predict charge flow behavior for rational GBMOF photocatalyst design. As for the S-scheme systems, this system is constructed through a staggered arrangement of a reduction-type semiconductor (usually g-C_3_N_4_), characterized by a smaller work function and a higher Fermi level, and an oxidation-type semiconductor (MOF), which possesses a larger work function and a lower Fermi level.^66,86–88^ This design promotes efficient e^−^/h^+^ separation while preserving high redox strength, making it highly effective for photocatalytic applications.^66,86,88–93^ Moreover, in this charge-carrier migration pathway, the band bending and the internal electric field at the 2D/3D interface further drive the selective recombination of low-energy carriers while retaining the high-energy redox pairs.^66,88^ In both cases, the graphene-based component usually serves as an electron mediator or transport bridge, accelerating charge migration and enhancing spatial separation.^84^ These charge-carrier transfer pathways directly dictate ROS generation and pollutant degradation efficiency, as discussed in the next section.

## 6. Photocatalytic degradation performance evaluation

In this section, the photocatalytic performance of various GBMOFs for the degradation of different dye pollutants is discussed to highlight recent advances, limitations, and key insights. In addition, key data such as degradation time, catalyst loading, initial dye concentration, the main ROS facilitating the degradation, pH, and the light source, are summarized in Table 2 alongside the photocatalytic degradation efficiency as percentages.

**Table 2:** Overview of the photocatalytic performance of graphene-/graphitic carbon nitride-based MOF materials for dye pollutant degradation^a^

GBMOF		Band gap (eV)	Dye pollutant	Light source	Experimental parameters	Degradation efficiency (%)	Time (min)	Main ROS	Ref.
Graphene/graphitic carbon nitride derivative	MOFs derivative								
GO	MIL-101(Fe)/PANCMA	NA	RhB	16 W UV lamp	Dose = 4 mg, pH = 6–8	100	60	˙OH	94
g-C_3_N_4_	Cu(ii) MOFs	1.91	MB	Visible light + H_2_O_2_	pH = 6, conc. = 10 mg L^−1^, dose = 200 mg	92.0	20	h^+^	68
g-C_3_N_4_	Cu(ii) MOFs	1.91	MG	Visible light + H_2_O_2_	pH = 6, conc. = 30 mg L^−1^, dose = 200 mg	92.9	35	—	68
g-C_3_N_4_	Cu(ii) MOFs	1.91	CR	Visible light + H_2_O_2_	pH = 6, conc. = 100 mg L^−1^, dose = 200 mg	98.2	3	h^+^ and ˙O_2_^−^	68
g-C_3_N_4_	Cu(ii) MOFs	1.91	MV	Visible light + H_2_O_2_	pH = 6, conc. = 10 mg L^−1^, dose = 200 mg	92.0	69	—	68
GO	Cu(ii) MOFs	2.04	MB	Visible light + ultrasonic	pH = 8, conc. = 10 mg L^−1^, dose = 40 mg	91.2	15	˙O_2_^−^ and ˙OH	105
g-C_3_N_4_/CdS	Ti-MOF	2.32	RhB	300 W Xe lamp	NA	90.2	90	˙O_2_^−^ and h^+^	74
rGO/TiO_2_	UiO-66	2.2	RhB	500 W Xe lamp	Dose = 15 mg, conc. = 10 mg L^−1^	>90	28	—	80
rGO/TiO_2_	Cu-BTC	2.3	RhB	500 W Xe lamp	Dose = 15 mg, conc. = 10 mg L^−1^	>90	>35	—	80
rGO/TiO_2_	ZIF-8	2.7	RhB	500 W Xe lamp	Dose = 15 mg, conc. = 10 mg L^−1^	>90	>35	—	80
g-C_3_N_4_/Ce	MIL-101(Fe)	2.34	MB	300 W Xe lamp	Dose = 15 mg, conc. = 15 mg L^−1^	88.17	75	˙O_2_^−^ and h^+^	69
g-C_3_N_4_/Ce	MIL-101(Fe)	2.34	RhB	300 W Xe lamp	Dose = 15 mg, conc. = 15 mg L^−1^	90.36	75	˙O_2_^−^ and h^+^	69
g-C_3_N_4_	Cu(ii) MOFs	2.63	MB	Visible light + H_2_O_2_	pH = 5, dose = 300 mg, conc. = 10 mg L^−1^	96.2	15	h^+^ and ˙OH	71
g-C_3_N_4_	Cu(ii) MOFs	2.63	MO	Visible light + H_2_O_2_	pH = 5, dose = 300 mg, conc. = 30 mg L^−1^	87.0	45	h^+^	71
g-C_3_N_4_	Cu(ii) MOFs	2.63	RhB	Visible light + H_2_O_2_	pH = 5, dose = 300 mg, conc. = 10 mg L^−1^	92.3	60	˙OH	71
g-C_3_N_4_	UiO-66	2.57	RhB	500 W Xe lamp	Dose = 20 mg, conc. = 10 mg L^−1^	∼98	180	˙O_2_^−^, ˙OH and h^+^	73
rGO	Cu-MOF@Ag_3_VO_4_	NA	Acid blue 92	Visible light	Dose = 10 mg, conc. = 10 mg L^−1^	>75	120	˙O_2_^−^	81
g-C_3_N_4_	UiO-66	2.72	MB	350 W Xe lamp	Dose = 50 mg, conc. = 10 mg L^−1^	100	240	˙O_2_^−^	70
g-C_3_N_4_	MIL-88A	2.29	Acid red 1	500 W Xe lamp	Dose = 10 mg, conc = 10 mg L^−1^	100	30	h^+^	78
GO	Cu/Cd MOF	2.7	MO	300 W Xe lamp	pH = 9, dose = 30 mg, conc. = 10 mg L^−1^	85.4	40	—	100
GO	Cu/Cd MOF	2.7	CR	300 W Xe lamp	pH = 9, dose = 30 mg, conc. = 10 mg L^−1^	90.1	40	—	100
GO	Cu/Cd MOF	2.7	MB	300 W Xe lamp	pH = 9, dose = 30 mg, conc. = 10 mg L^−1^	92.0	40	˙O_2_^−^	100
GO	Cu/Cd MOF	2.7	RhB	300 W Xe lamp	pH = 9, dose = 30 mg, conc. = 10 mg L^−1^	79.6	40	—	100
g-C_3_N_4_	UiO-66	2.92	RhB	300 W Xe lamp	Dose = 20 mg, conc. = 60 mg L^−1^	∼100	70	˙O_2_^−^	72
g-C_3_N_4_	MIL-101(Fe)	2.05	RhB	100 W LED lamp + H_2_O_2_	pH = 4.8, dose = 50 mg, conc. = 10 mg L^−1^	99.3	90	˙O_2_^−^, ˙OH and e^−^/h^+^	77
g-C_3_N_4_	MIL-101(Fe)	1.97–2.88	RhB	150 W Xe lamp + H_2_O_2_	Dose = 200 mg, conc. = 50 mg L^−1^	100	240	˙OH and e^−^/h^+^	111
g-C_3_N_4_	MIL-53(Fe)	NA	RhB	300 W Xe lamp	Dose = 500 mg	100	75	—	112
g-C_3_N_4_	Ce-MOF	2.45	MB	LED light	Dose = 25 mg, conc. = 10 mg L^−1^	96.5	120	—	95
GO	Ag–Zn-BTC	2.48	RY 145	60 W fluorescent lamps	pH = 3, dose = 20 mg, conc. = 50 mg L^−1^	∼100	35	˙O_2_^−^ and h^+^	101
GO	Zn-BTC	3.06	RY 145	60 W fluorescent lamps	pH = 3, dose = 20 mg, conc. = 50 mg L^−1^	∼100	80	˙O_2_^−^ and h^+^	101
GO	Ag-BTC	2.13	RY 145	60 W fluorescent lamps	pH = 3, dose = 20 mg, conc = 50 mg L^−1^	∼100	60	˙O_2_^−^ and h^+^	101
g-C_3_N_4_	Ag_3_PO_4_/Zr-BDC	2.91	MB	85 W tungsten lamp	Dose = 75 mg, pH = 6, conc. = 10 mg L^−1^	95	240	h^+^	75
g-C_3_N_4_	Ag_3_PO_4_/Zr-BDC	2.91	MB	Natural sunlight	Dose = 75 mg, pH 6, conc. = 10 mg L^−1^	93	105	h^+^	75
g-C_3_N_4_	Zr-MOF	2.97	MB	85 W tungsten lamp	Dose = 75 mg, pH = 6, conc. = 10 mg L^−1^	80	240	h^+^	75
rGO	MOF-5	NA	MO	Xe solar simulator	Dose = 20 mg, conc. = 10^−5^ M	92	20	˙O_2_^−^, ˙OH and 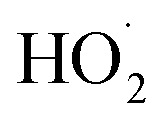	82
rGO	MOF-5	NA	RhB	Xe solar simulator	Dose = 20 mg, conc. = 10^−5^ M	97	20	˙O_2_^−^, ˙OH and 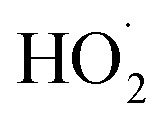	82
rGO	MOF-5	NA	MB	Xe solar simulator	Dose = 20 mg, conc. = 10^−5^ M	93	20	˙O_2_^−^, ˙OH and 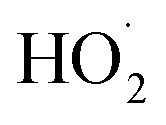	82
GQD	UiO-66-NH_2_	NA	MB	400 W metal-halide light	Conc. = 40 mg L^−1^	98.6	180	˙O_2_^−^	106
g-C_3_N_4_@ZnFe_2_O_4_	UiO-66	2.8	RhB	Visible light	NA	98	60	˙O_2_^−^, ˙OH and h^+^	113
GO	Starch@ZIF-67	NA	Acid blue 92	LED-visible light	Dose = 8 mg, pH = ∼7, conc. = 40 mg L^−1^	86.4	180	˙O_2_^−^	114
g-C_3_N_4_	CoTiO_3_-ZIF-67	2.36–2.64	MO	300 W Xe lamp	Dose = 70 mg, conc. = 10 mg L^−1^	>90	240	˙O_2_^−^	115
N-doped g-C_3_N_4_	NH_2_-MIL-125(Ti)	2.43–2.61	RhB	300 W Xe lamp	Dose = 20 mg, conc = 10 mg L^−1^	96.4	120	˙O_2_^−^ and h^+^	88
g-C_3_N_4_	NH_2_-MIL-53(Fe)	2.62	RhB	300 W Xe lamp	Dose = 10 mg, conc. = 60 mg L^−1^	95	80	˙OH	116
GO	MIL-53(Fe)@BiVO_4_	2.20	RhB	500 W Xe lamp	Dose = 10 mg, conc. = 15 mg L^−1^	>80	60	˙O_2_^−^, and ˙OH	117
g-C_3_N_4_	MIL-125(Ti)	3.24	RhB	300 W Xe lamp	Dose = 40 mg, conc. = 50 mg L^−1^	95.2	60	˙O_2_^−^, and h^+^	118
GO@BiOBr	MOF-5	2.1	RhB	Simulated solar light	NA	92	120	˙O_2_^−^	119
rGO	NiCu-MOF	2.89	Orange G	300 W Xe lamp	Dose = 30 mg, pH = 6	93.5	60	˙O_2_^−^, and ˙OH	83
rGO	NiCu-MOF	2.89	RhB	300 W Xe lamp	Dose = 30 mg, pH = 6	∼97.8	60	˙O_2_^−^, and ˙OH	83
rGO	N-doped ZIF-8	2.8	MB	300 W solar simulator	pH = 8, dose = 400 mg, conc. = 10 mg L^−1^	99	60	˙O_2_^−^	97
GQD	MIL-100(Fe)	1.93	CR	400 W LED lamp	Dose = 25 mg, pH = 7, conc. = 50 mg L^−1^	93.8	5	˙O_2_^−^ and h^+^	120
GO	Starch/MIL88A	NA	Acid blue 92	LED lamp	pH ∼7, dose = 4 mg, conc. = 225 mg L^−1^	98.5	105	˙O_2_^−^, and ˙OH	121
GO@chitosan	Cu_3_(btc)_2_	2.75	MB	500 W Xe lamp + H_2_O_2_	pH = 7, dose = 500 mg, conc. = 10 mg L^−1^	98	60	˙O_2_^−^, and ˙OH	122
g-C_3_N_4_@ZnO	MOF	2.73	RhB	Xe lamp	Dose = 40 mg, conc. = 20 mg L^−1^	99.09	150	˙O_2_^−^	123
GO	MOF@Bi_2_O_3_	2.46	RhB	500 W Xe lamp	Dose = 50 mg, conc. = 10 mg L^−1^	>85	60	˙O_2_^−^, and ˙OH	124
g-C_3_N_4_	MIL-88A	NA	RhB	1000 W iodine tungsten lamp	Dose = 100 mg, pH = 7, conc. = 10 mg L^−1^	100	30	˙O_2_^−^, and ˙OH	125
g-C_3_N_4_	MIL-125(Ti)@sodium alginate	NA	RhB	300 W Xe lamp	pH = 5, conc. = 30 mg L^−1^	99.23	120	˙O_2_^−^, and h^+^	126
g-C_3_N_4_	MIL-125(Ti)@sodium alginate	NA	MO	300 W Xe lamp	pH = 5, conc. = 30 mg L^−1^	81.65	120	˙O_2_^−^, and h^+^	126
g-C_3_N_4_	MIL-125(Ti)@sodium alginate	NA	MB	300 W Xe lamp	pH 5, conc. = 30 mg L^−1^	78.23	120	˙O_2_^−^, and h^+^	126
g-C_3_N_4_	MIL-88A	2.05–2.67	MB	300 W Xe lamp + H_2_O_2_	Dose = 10 mg	85	120	˙O_2_^−^, and ˙OH	108
g-C_3_N_4_	MIL-88A	2.05–2.67	MB	300 W Xe lamp	Dose = 10 mg	75	120	˙O_2_^−^, and ˙OH	108
GO	Cu-MOF@chitosan	NA	MO	6 W UV-C lamp	Dose = 750 mg, pH = 7, conc. = 30 mg L^−1^	>90	120	˙OH	127
g-C_3_N_4_	ZIF-8@AgI	2.6	MB	200 W LED lamps	pH = 6.7, dose = 100 mg, conc. = 30 mg L^−1^	94.2	120	˙O_2_^−^	98
g-C_3_N_4_	ZIF-8	2.72	MB	100 W LED lamps	pH = 6.7, dose = 100 mg, conc. = 30 mg L^−1^	85.9	120	˙O_2_^−^	98
rGO	Zn-MOF	NA	MB	100 W bulb	Dose = 40 mg, pH = 8, conc. = 10 mg L^−1^	98.6	60	˙O_2_^−^	128
rGO	ZIF-8	∼3.18	MB	300 W Xe lamp	Dose = 1000 mg, conc. = 10 mg L^−1^	82	120	—	129
rGO	MIL-125(Ti)	2.90	CR	32 W fluorescent light	Dose = 15 mg, conc. = 10 mg L^−1^	92.6	180	˙OH	96
g-C_3_N_4_@CdS	UiO-66(Ce)	2.34	RhB	85 W tungsten lamp	pH = 8, dose = 60 mg, conc. = 10 mg L^−1^	99.5	120	˙O_2_^−^, ˙OH and h^+^	130
GO@g-C_3_N_4_	NH_2_-MIL-88B(Fe)	2.0	Direct red 23	100 W LED lamp + H_2_O_2_	pH = 3, dose = 2 mg, conc. = 60 mg L^−1^	99	70	˙OH and h^+^	109
g-C_3_N_4_	Ni/MoS_2_/MOF-2	1.75	MB	Natural sunlight	Dose = 2 mg, conc. = 10 mg L^−1^	91	90	˙O_2_^−^, and ˙OH	102
g-C_3_N_4_	MoS_2_/MOF-2	NA	MB	Natural sunlight	Dose = 2 mg, conc = 10 mg L^−1^	88	90	˙O_2_^−^, and ˙OH	102
g-C_3_N_4_	NiS_2_/MOF-2	NA	MB	Natural sunlight	Dose = 2 mg, conc. = 10 mg L^−1^	81	90	˙O_2_^−^, and ˙OH	102
g-C_3_N_4_	MIL-53(Al)	2.6	RhB	250 W fluorescent lamp	Dose = 50, conc. = 10 mg L^−1^	∼100	75	˙O_2_^−^	131
rGO@Ag	MIL-125(Ti)	NA	RhB	300 W Xe lamp	Dose = 40 mg, conc. = 50 mg L^−1^	95.7	50	˙O_2_^−^	99
g-C_3_N_4_	Co-ZIF-67	NA	CV	Solar simulator	Dose = 20 mg, conc. = 4 mg L^−1^	95	80	˙O_2_^−^ and ˙OH	132
GO	MIL-88(Fe)	NA	MB	Natural sunlight	Dose = 50 mg, conc. = 100 mg L^−1^	∼100	20	—	103
GO	MIL-88(Fe)	NA	RhB	Natural sunlight	Dose = 50 mg	∼100	30	—	103
rGO	NH_2_-MIL-125(Ti)	NA	MB	300 W Xe lamp	Dose = 30 mg, conc. = 5.4 × 10^−5^ mol L^−1^	>98	25	˙O_2_^−^	133
GO@Fe_3_O_4_	Nd-MOF	2.39	MB	Visible light	Dose = 2 mg, conc. = 10 mg L^−1^, pH = 11	∼95	80	e^−^ and h^+^	134
GO	ZIF-8	3.60	AR88	40 W LED lamp	Dose = 15 mg, conc. = 60 mg L^−1^, pH = 5	77.2	90	˙O_2_^−^ and ˙OH	107
GO	ZIF-8	3.60	AR88	40 W LED lamp + ultrasonic	Dose = 15 mg, conc. = 60 mg L^−1^, pH = 5	85.4	90	˙O_2_^−^ and ˙OH	107
gC_3_N_4_/Fe_3_O_4_	Zn-MOF-5	1.45–3.78	RhB	Visible light	Dose = 2 mg, pH 9	95	90	˙OH	135
GO	ZIF-8/HKUST-1	3.73	CR	UV-LED lamp	Dose = 10 mg, pH = 7, conc. = 50 mg L^−1^	91.81	60	˙O_2_^−^ and ˙OH	136
g-C_3_N_4_	NH_2_-MIL-101(Fe)	NA	MO	300 W Xe arc lamp	Dose = 20 mg, conc. = 20 mg L^−1^	78.0	100	—	137
GO	MOF@CuO	2.14	MB	Natural sunlight	Dose = 5 mg, pH = 7, conc. = 10 mg L^−1^	99.92	30	˙O_2_^−^ and ˙OH	104
GO	MOF-5	3.5	MB	Solar light	pH = 6.8, dose = 20 mg, conc. = 10 mg L^−1^	92	390	˙O_2_^−^, ˙HO_2_, and ˙OH	138
GO@sodium alginate	NH_2_-MIL-88B(Fe)	2.31	MB	300 W Xe lamp	Conc. = 10 mg L^−1^	99.67	60	˙O_2_^−^, and h^+^	139
g-C_3_N_4_	MOF-5	2.72	RhB	Natural sunlight	Dose = 30 mg, conc. = 10 mg L^−1^, pH = 13	91.5	90	˙O_2_^−^	110
GO	MIL-100(Fe)	2.35	MB	300 W Xe lamp	Dose = 100, conc. = 200 µmol L^−1^	95	210	˙OH	140
g-C_3_N_4_	Ni-MOF	NA	RhB	Natural sunlight	Dose = 20 mg, pH = 7	∼93	120	˙O_2_^−^ and ˙OH	85

a NA = not available.

**Fig. 8:**
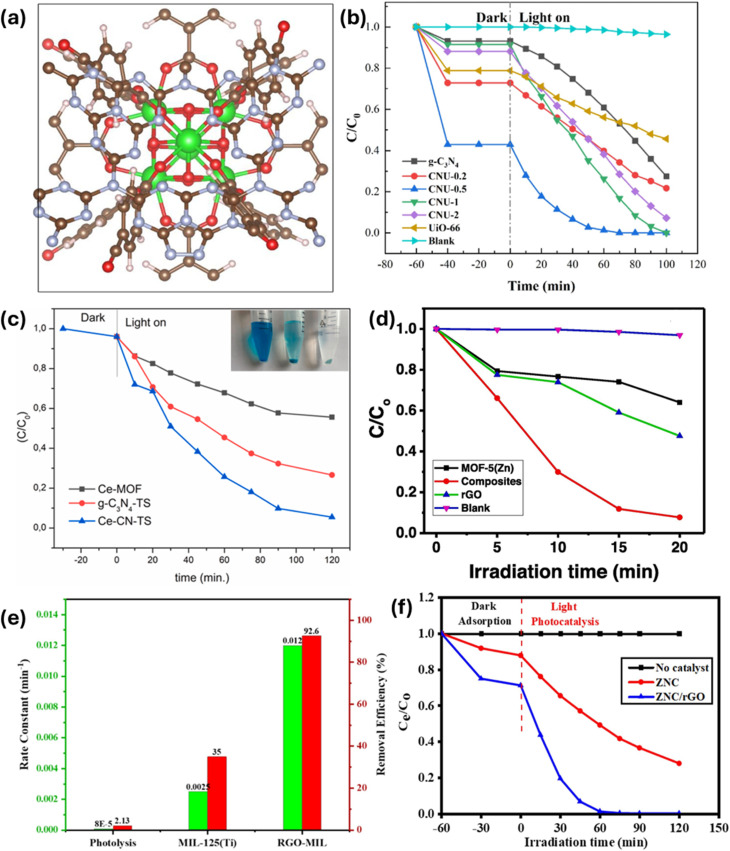
(a) DFT configuration of g-C_3_N_4_ on a UiO-66 surface and (b) degradation performance of g-C_3_N_4_@UiO-66 for RhB. Reproduced from ref. 72 with permission from Elsevier B.V., D. Lan, H. Zhu, J. Zhang, C. Xie, F. Wang, Y. Zheng, Z. Guo, M. Xu and T. Wu, *Appl. Surf. Sci.*, 2024, **655**, 159623, copyright 2024. (c) Degradation rate of MB by g-C_3_N_4_@Ce-MOF.^95^ Reproduced from Durmus *et al.*, 2023, licensed under CC BY. Open access, which permits unrestricted use, distribution, and reproduction in any medium. (d) Degradation performance of rGO@MOF-5 for MB. Reproduced from ref. 82 with permission from Elsevier B.V., D. Lan, Q. V. Thi, M. S. Tamboli, Q. Thanh Hoai Ta, G. B. Kolekar and D. Sohn, *Mater. Sci. Eng. B*, 2020, **261**, 114678, copyright 2020. (e) Degradation performance of rGO@MIL-125(Ti) for CR. Reproduced from ref. 96 with permission from Elsevier B.V., D. Lan, R. Fatima, M. N. Afridi, I. Mohdeb, P. Madhusudan and Y. Hwang, *J. Water Process Eng.*, 2025, **69**, 106893, copyright 2024. (f) Degradation performance of N-doped rGO@ZIF-8 for MB. Reproduced from ref. 97 with permission from Elsevier B.V., M. M. Kaid, O. Elbanna, S. A. El-Hakam, H. M. El-Kaderi and A. A. Ibrahim, *J. Photochem. Photobiol., A*, 2022, **430**, 114001, copyright 2022.

For example, in a particular case study,^94^ the comparative photocatalytic performance of GO/PANCMA, MIL-101(Fe)/PANCMA, and GO/MIL-101(Fe)/PANCMA nanofibers for the degradation of RhB was explored. It was observed that in under 60 minutes of UV light irradiation, 100% degradation was achieved by GO/MIL-101(Fe)/PANCMA in contrast to MIL-101(Fe)/PANCMA, which only degraded <75%, and GO/PANCMA, which showed no degradation efficiency under the same experimental conditions. The excellent RhB degradation efficiency exhibited by GO/MIL-101(Fe)/PANCMA compared to MIL-101(Fe)/PANCMA was attributed to the incorporation of GO, which boosted electron transfer efficiency during the photocatalytic degradation process. Moreover, the formation of heterostructures between GO and the MOF, as well as the existence of more active surface reaction sites, enhances the dye photocatalytic degradation efficiency. A notable finding here was that GBMOF containing 0.025 wt% GO gave the best result, and amounts higher or lower than that did not give a satisfactory degradation result.^94^ Similarly, when g-C_3_N_4_/Ce@MIL-101(Fe) was employed for the photocatalytic degradation of MB and RhB, over 88% efficiency was achieved in 75 minutes. It was stated that the addition of Ce/g-C_3_N_4_ effectively decreases the overall band gap value of g-C_3_N_4_/Ce@MIL-101(Fe), which then enhances visible-light absorption, and thus boosts the photocatalytic degradation efficiency. Nevertheless, it was noted that when the amount of g-C_3_N_4_/Ce was augmented to 5 mmol, the band gap value increased slightly, which is ascribed to the MOF surface being covered by too much g-C_3_N_4_/Ce, which obstructs the GBMOF light absorption and decreases the photocatalytic degradation activity/efficiency.^69^ One key insight is that optimizing GBMOF performance requires careful consideration of the trade-off between GO/g-C_3_N_4_ loading and MOF composition. Similar patterns of improved dye photocatalytic degradation performance by GBMOFs as against individual components were also documented in other case studies, as displayed in Fig. 8.^72,82,95–97^

**Fig. 9:**
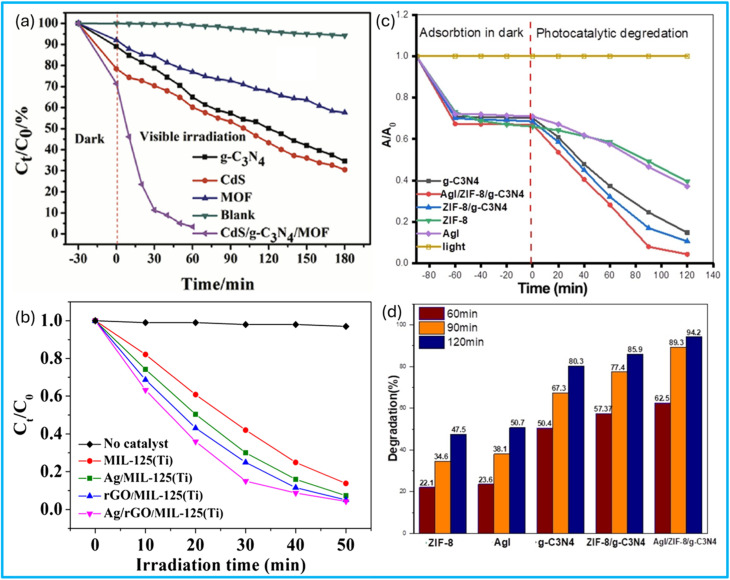
Photocatalytic degradation performance of ternary (a) CdS/g-C_3_N_4_/MOF. Reproduced from ref. 74 with permission from Elsevier B.V., Y. Chen, B. Zhai, Y. Liang, Y. Li and J. Li, *J. Solid State Chem.*, 2019, **274**, 32–39, copyright 2019. (b) rGO@Ag@MIL-125(Ti). Reproduced from ref. 99 with permission from John Wiley and Sons, X. Yuan, H. Wang, Y. Wu, G. Zeng, X. Chen, L. Leng, Z. Wu and H. Li, *Appl. Organomet. Chem.*, 2016, **30**, 289–296, copyright 2016. (c and d) g-C_3_N_4_@ZIF-8@AgI as against binary/individual components. Reproduced from ref. 98 with permission from Elsevier B.V., H. H. Abed and S. H. Ammar, *Inorg. Chem. Commun.*, 2024, **169**, 113056, copyright 2024.

Recent advances include the design and application of ternary GBMOF nanocomposites. In one instance, Tang *et al.*^80^ explored the photocatalytic performance of rGO@TiO_2_@Cu-BTC, rGO@TiO_2_@UiO-66, and rGO@TiO_2_@ZIF-8 for the degradation of RhB. It was discovered that, after treatment with a 500 W xenon lamp for less than 35 minutes, a degradation efficiency of over 90% was recorded for all three ternary GBMOFs, which surpasses that of bare TiO_2_, Cu-BTC, rGO@TiO_2_, UiO-66, and ZIF-8 under the same conditions. This shows the positive impact of the ternary composite. In this study, the ternary GBMOF composites were able to deliver excellent photocatalytic degradation performance because of their lower band gap values compared to individual components, better photon scattering-adsorption, and synergistic effects where TiO_2_ plays a primary role as a photocatalyst while rGO and MOF function as the electron acceptor and carrier for the dye pollutants, respectively. One significant revelation here is that microporous GBMOFs are favourable for adsorbing dye pollutants of small molecular structures compared to mesoporous GBMOFs. Moreover, GBMOF ligands and the RhB functional group have a strong coordinated π–π interaction, which leads to a stronger attractive coordination force between the GBMOF and the dye pollutant molecule. This attractive force was able to trap the RhB molecules at the surface of the GBMOF, where the photogenerated radicals can carry out adequate degradation attacks. A study on incident photon-to-electron conversion efficiency also established that the strong chemical connection between the TiO_2_ and rGO is a crucial precondition for the improved photocatalytic degradation performance of GBMOF.^80^ For future studies, when it comes to ternary GBMOF composites, it is also recommended that metallic/metal oxide nanoparticles should be loaded on the rGO/g-C_3_N_4_ component rather than directly on the MOFs to avoid the blockage of the MOFs' porous structure. Moreover, because GO/rGO/g-C_3_N_4_ provides a large, planar, defect-rich surface with abundant coordinated anchoring sites (*e.g.*, residual oxygenated groups), loading metallic/metal oxide nanoparticles on it will enable uniform nanoparticle dispersion and prevent nanoparticles from agglomerating, which is a potential problematic issue. A similar trend of photocatalytic degradation performance was recorded for g-C_3_N_4_@ZIF-8@AgI,^98^ CdS/g-C_3_N_4_/Ti-MOF,^74^ and rGO@Ag@MIL-125(Ti),^99^ as shown in Fig. 9, and other ternary GBMOFs, as presented in Table 2, where ternary GBMOFs performed better than the binary and single-component counterparts. According to the findings, the strong synergetic absorption of visible light, indirect dye photosensitization, and reduced band gap brought by heterojunction formation enhance optical response and enable visible light to supply adequate energy for the mobility of electrons from the VB to the CB.^74,98^

**Fig. 10:**
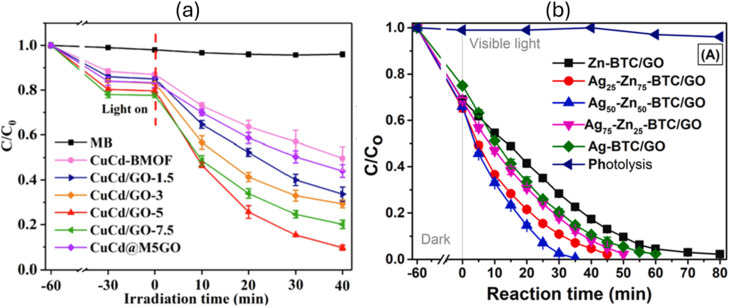
Photocatalytic degradation performance of bimetallic GBOMOFs: (a) GO@CuCd-MOF for MB. Reproduced from ref. 100 with permission from Elsevier B.V., J. Chen, J. Wei, H. Zhang, X. Wang, L. Fu and T. H. Yang, *Appl. Surf. Sci.*, 2022, **594**, 153493, copyright 2022. (b) GO@Ag–Zn-BTC for RY 145 dye in water. Reproduced from ref. 101 with permission from Elsevier B.V., M. B. Nguyen, G. H. Le, T. D. Nguyen, Q. K. Nguyen, T. T. T. Pham, T. Lee and T. A. Vu, *J. Hazard. Mater.*, 2021, **420**, 126560, copyright 2021.

Also, recently, research on GBMOFs has progressed to bimetallic metal–organic frameworks due to their synergistic effects in enhancing active sites and charge transfer. For instance, Chen *et al.* explored the photocatalytic efficiency of a GO@Cu/Cd bimetallic MOF for the degradation of four dyes (MO, CR, MB, and RhB) under a 300 W xenon lamp exposure. Their findings revealed that in 40 minutes, over 80% degradation efficiency was achieved for all dyes, which was higher than that achieved by bare Cu/Cd bimetallic MOF that exhibited less than 50% degradation efficiency. The improved photocatalytic activity can be rationalized through the formation of metal–semiconductor heterojunctions. Specifically, the bimetallic nodes (*e.g.*, Cu/Cd clusters) often exhibit metallic character due to their d-orbital electrons and redox-active behavior. Upon close contact with GO, Fermi-level equilibration occurs, leading to the development of a Schottky barrier that promotes directional charge-carrier migration. Furthermore, it was opined that the strong H-bonding that is formed by coordinated water molecules, carboxylate, and phosphonate groups in the CuCd bimetallic MOF with carboxyl functional groups on GO, establishes enhanced channels of fast charge transfer, which then improves dye photocatalytic degradation output.^100^ These findings are in very good agreement with what was exhibited by rGO@NiCu-MOF for the degradation of Orange G and RhB. The remarkable degradation efficiency (>93% in 60 minutes) was attributed to the introduction of rGO, which led to the formation of a Z-scheme heterojunction, rather than a simple type-II alignment.^83^ As presented in Table 2, a similar pattern of excellent degradation performance was observed for the photocatalytic degradation of RY 145, where GO@Ag–Zn-BTC outshone GO@Zn-BTC and GO@Ag-BTC under the same experimental conditions.^101^ One key insight here is that the simultaneous presence of two metal nodes opens up more photoactive sites for improved light striking and photon generation and also optimizes the overall band alignment and interfacial charge transfer compared to single-metal analogues. More specifically, beyond conventional semiconductor heterojunction effects, these studies demonstrate that bimetallic GBMOFs exhibit metal–semiconductor interfacial interactions, where metal nodes act as electron trapping sites, and when coupled with graphene-based materials, which provide rapid electron transport pathways, these synergistically enhance charge separation as well as photocatalytic degradation performance. Nevertheless, it was observed that excess GO, rGO, or Ag undermines the synergistic cooperation between GO/rGO and the bimetallic MOF, which is ascribed to the active site shield effect (blockage of active site) and obstruction of charge transfer channels. This is evident in Fig. 10a and b, where GO@bimetallic MOF containing moderate GO (5%) and Ag (50%) performed better.^100,101^

In line with the principle of green chemistry, photocatalytic degradation research has advanced to the use of natural sunlight instead of traditional stimulated light for energy efficiency. In a particular case study on the photocatalytic degradation of MB by g-C_3_N_4_@Ag_3_PO_4_/Zr-BDC, the performance of an 85 W tungsten lamp against natural sunlight was evaluated. Amazingly, as shown in Table 2, natural sunlight exhibited a superior (>90%) degradation efficiency in a shorter time of 105 minutes in comparison to a tungsten lamp, which delivers >90% in 240 minutes.^75^ In a comparable study, g-C_3_N_4_@Ni/MoS_2_/MOF-2, g-C_3_N_4_@MoS_2_/MOF-2, and g-C_3_N_4_@NiS_2_/MOF-2 also achieved over 80% MB dye degradation efficiency under natural sunlight within 90 minutes,^102^ and this performance trend under natural sunlight is consistent with what was recorded for GO@MIL-88(Fe)^103^ and GO@MOF@CuO,^104^ as presented in Table 2. This suggests that GBMOFs have potential for pilot-scale photocatalytic degradation of dye pollutants, even though inconsistent photocatalytic performance across different geographical locations, weather conditions, and day–night cycles has not yet been taken into consideration, as this may complicate reliable scale-up.

Another recent advancement is in the use of GBMOFs under an assisted photocatalytic degradation system. For example, in a case study on the degradation of MB by GO@Cu(ii) MOFs, 92.7% efficiency was recorded in a photocatalytic scenario. However, when the photocatalytic system was assisted by ultrasound, an enhanced degradation efficiency of 94.6% was achieved under the same experimental conditions, which shows the impact of the assisted photocatalytic degradation system. Findings also revealed that in any case, GO@Cu(ii) MOF outperforms both GO and Cu(ii) MOF individually.^105^ From our own perspective, it can be deduced that the synergetic activities of sono-photocatalysis circumvent the deposition of MB dye degradation intermediate products on the surface of GO@Cu(ii) MOF and thus promote more active-site availability and enhance mass transfer in solution. In addition, more radicals were synergistically generated by photo-assisted cavitation bubble-driven sonoluminescence on the heterogeneous GBMOF material upon bubble collapse, creating a reliable and effective degradation system for the MB dye. Also, when graphene quantum dot@UiO-66-NH_2_ was used for membrane photocatalytic degradation of MB under metal-halide light illumination, 98.6% efficiency was obtained in 180 minutes. The findings demonstrated that the MOF enhances impurity trapping in addition to facilitating MB transit in aqueous solution with the membrane fiber framework.^106^ In a recent study, as presented in Table 2, a piezo-assisted photocatalytic system exhibited superior AR88 degradation compared to the non-assisted system using GO@ZIF-8 due to improved radical generation aided by ultrasonic force.^107^ A key potential limitation from our perspective lies in the energy efficiency of such piezo-assisted photocatalytic systems, as reliance on continuous ultrasonic input may increase operational costs and limit practical large-scale wastewater treatment applications, despite the observed enhanced AR88 degradation. In another assisted photocatalytic study,^68^ the degradation of MB under visible light using g-C_3_N_4_@Cu(ii) MOFs was assisted by H_2_O_2_. The findings showed that at a hydrogen peroxide concentration of 50.0 mM, an improved degradation efficiency of 91.0% was achieved. However, the degradation rate dropped to 88.3% when H_2_O_2_ was introduced in excess, at a concentration of 70.0 mM, because too much hydrogen peroxide scavenged the ˙OH. Conversely, only ∼22.2% of MB was degraded in the absence of H_2_O_2_ as an electron acceptor because of the insufficient production of ˙OH radicals. It was also noted that the degradation output at acidic pH (<6) was low due to strong repulsion between GBMOF and the cationic MB dye, resulting from high protonation of the g-C_3_N_4_@Cu(ii) MOF surface.^68^ This suggests that g-C_3_N_4_@Cu(ii) MOF might encounter efficiency issues in a real, harsh acidic environment. In contrast, the g-C_3_N_4_@MIL-101(Fe) photocatalytic system assisted by H_2_O_2_ delivered over 99% RhB degradation in both acidic and basic environments.^77^ The excellent photocatalytic degradation performance can be attributed to the Fenton-like contribution from the MOF(Fe) in GBMOF(Fe), which is not possible for g-C_3_N_4_@Cu(ii) MOF, leading to dual assistance. This is consistent with what was documented for the degradation of MB and Direct red 23 by g-C_3_N_4_@MIL-88A^108^ and GO@g-C_3_N_4_@NH_2_-MIL-88B(Fe)^109^ in a H_2_O_2_-assisted photocatalytic degradation system. Thus, it is recommended that future researchers should begin to look into dual-assisted photocatalytic degradation-oriented technology using advanced functional materials such as GBMOFs, especially those with inherent Fenton properties.

Interestingly, despite the recent advances reported in the foregoing paragraphs, degradation efficiency alone is not an adequate metric for comparing photocatalysts because it depends heavily on experimental parameters. Thus, more appropriate metrics, such as quantum efficiency, mineralization efficiency, and TOC removal, are needed to complement degradation efficiency.

For instance, in a particular case study, 100% degradation efficiency was achieved in 240 minutes for MB using g-C_3_N_4_@UiO-66. However, only 72% TOC removal was accomplished in 240 min, indicating incomplete mineralization of MB. Comparably, when N-doped g-C_3_N_4_@NH_2_-MIL-125(Ti) and g-C_3_N_4_@MOF-5 were employed, over 90% RhB degradation was recorded, while the TOC removal was just 49% and 82%, respectively.^88,110^ This discrepancy in the degradation and TOC removal efficiency is because some organic intermediates, such as carboxylic acids and aldehydes, persisted when the chromophores (aromatic rings) were completely broken.^70,88^ This suggests that while degradation proceeds effectively, the conversion of these intermediates into CO_2_ and H_2_O remains a limiting step in the overall photocatalytic process.^70,88^ Moreover, one critical insight here is that high degradation output does not always translate to high/complete mineralization of the dye pollutant, which is the fundamental aim of photocatalytic degradation.

Other essential metrics for assessing photocatalytic performance are apparent quantum yield, which measures the number of dye molecules degraded per number of photons utilized, and catalyst surface efficiency, which measures the number of degraded dye molecules per unit surface area of the photocatalyst. In recent research by Fatima *et al.*^96^ on the photocatalytic degradation of CR, rGO@MIL-125(Ti) displayed a higher surface efficiency than ordinary MIL-125(Ti), demonstrating that the GBMOF is a more effective photocatalyst material for the degradation of CR. In contrast, rGO@MIL-125(Ti) exhibited a promising quantum yield of 4.61 × 10^−7^ molecules per photons but is less competitive with other photocatalytic materials such as Ag–TiO_2_ (7.46 × 10^−8^), Fe–TiO_2_ (8.05 × 10^−7^), and ZnO (6.01 × 10^−8^). One major takeaway here is that while the degradation efficiency is satisfactory (>90%), it does not automatically mean excellent or competitive quantum efficiency.^96^ Thus, it is recommended that future studies should work on GBMOFs that can deliver both satisfactory degradation and quantum efficiency. In addition, looking at the literature, quantum efficiency, mineralization efficiency, and TOC removal are underexplored or often overlooked, constituting a critical gap that needs attention from future researchers for the holistic evaluation of GBMOFs photocatalytic performance.

## 7. Recoverability, reusability, and stability dynamics

**Fig. 11:**
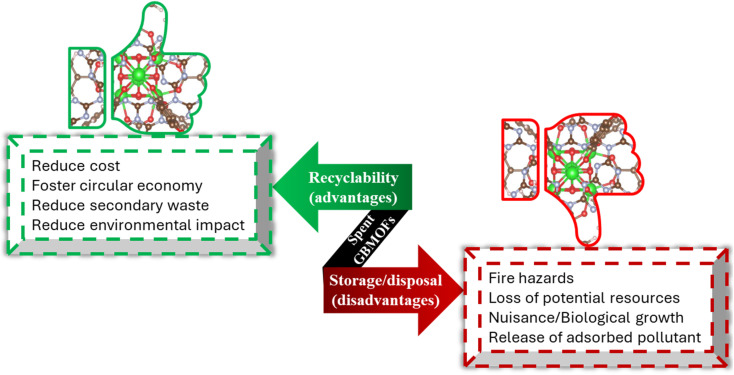
Benefits of reusing spent GBMOFs and dangers associated with their disposal or storage. Reproduced from ref. 144 with permission from Elsevier B.V., C. T. Umeh, A. B. Akinyele, N. H. Okoye, S. S. Emmanuel, K. O. Iwuozor, I. P. Oyekunle, J. O. Ocheje and J. O. Ighalo, *Environ. Nanotechnol., Monit. Manage.*, 2023, **20**, 100891, copyright 2023.

The proof of industrial applicability of any material and technique in water and wastewater remediation is heavily dependent on its alignment with the circular, green, blue, and zero-waste economy. Thus, a study on the recoverability, reusability, and stability potential of GBMOFs in the photocatalytic degradation of dye pollutants is non-negotiable. As summarized in Fig. 11, aside from waste mitigation, the recoverable/reusable photocatalyst also lowers overall treatment costs.^141–144^ Moreover, from pilot scale, ecological, and practical perspectives, material recoverability/reusability efficiency is essential, as it helps in designing sustainable water treatment technology.^144,145^Table 3 presents a summary of the reusability efficiency of various GBMOFs for dye photocatalytic degradation.

**Table 3:** Summary of the reusability performance of graphene-/graphitic carbon nitride-based MOF materials for dye pollutant degradation^a^

GBMOF		Specific surface area (m^2^ g^−1^)	Dye	Eluting agent	% Degraded in the 1st cycle	No. of cycles	% Degraded after last cycle	Ref.
GO	MIL-101(Fe)	3.6273	RhB	Filtration	93.7	30	73	94
g-C_3_N_4_/CdS	MOF	238.43	RhB	—	∼90.2	3	∼90	146
g-C_3_N_4_	Cu-MOF	NA	MB	—	92	5	81.3	68
g-C_3_N_4_	MIL-101(Fe)	113.41	RhB	—	90.36	5	86	69
g-C_3_N_4_	Cu(ii) MOF	NA	MB	—	96.2	5	90	71
g-C_3_N_4_	UiO-66	384	MB	EtOH + H_2_O	100	5	>80	70
g-C_3_N_4_	MIL-88A	NA	Acid red 1	—	97	5	88.6	78
GO	CuCd-BMOF	131.3	MB	Ultrapure water	92	4	>87.4	100
g-C_3_N_4_	UiO-66	386.19	RhB	—	∼100	5	95.43	72
g-C_3_N_4_	MIL-101(Fe)	35.1	RhB	Centrifugation + EtOH	99.35	5	97.96	77
g-C_3_N_4_	Ce-MOF	83	MB	Water + EtOH	96.5	4	80.5	95
GO	Ag–Zn-BTC	1519	Reactive yellow 145	—	100	4	>90	101
g-C_3_N_4_	Zr-BDC	449	MB	Centrifugation	95.1	3	92.9	75
rGO	MOF-5	NA	MB	Centrifugation + distilled water	>90	4	>72	82
rGO	MOF-5	NA	RhB	Centrifugation + distilled water	>95	4	>90	82
rGO	MOF-5	NA	MO	Centrifugation + distilled water	90		80	82
g-C_3_N_4_	ZnFe_2_O_4_/UiO-66	121.9	RhB	—	>90	6	<80	113
GO	ZIF-67	NA	Acid blue 92	—	86.4	3	82	114
g-C_3_N_4_	ZIF-67@TiO_2_	NA	MO	—	>90	3	<90	115
g-C_3_N_4_	NH_2_-MIL-125(Ti)	565.24	RhB	—	96.4	3	>85	88
g-C_3_N_4_	NH_2_-MIL-53(Fe)	99.4	RhB	Centrifugation + deionized water	95	4	90	116
GO	BiVO_4_/MOF	33.440	RhB	Centrifugation	∼80	4	∼75	117
g-C_3_N_4_	MIL-125(Ti)	328.0	RhB	Centrifugation	95.2	5	∼91.7	118
GO	BiOBr/MOF-5	184.95	RhB	—	92	4	<92	119
rGO	NiCu@isonicotinic acid	457.5	RhB	Water + EtOH	97.8	5	∼93	83
rGO	NiCu@isonicotinic acid	457.5	Orange G	Water + EtOH	93.5	5	∼84	83
rGO	ZIF-8	1412.2	MB	Deionized water + MeOH	98.9	5	93.2	97
GQD	MIL-100(Fe)	1878	CR	Centrifugation + water	98.3	4	90.1	120
GO	MIL88A	NA	Acid blue 92	Centrifugation + EtOH	98.5	5	95	121
GO–CS	Cu_3_(btc)_2_	947	MB	Deionized water	98	5	>75	122
g-C_3_N_4_	MIL-88A	38.231	RhB	—	∼100	6	∼100	125
g-C_3_N_4_	MIL-125(Ti)/sodium alginate	NA	RhB	—	99.23	7	85	126
g-C_3_N_4_	MIL-88A	15.52	MB	—	∼55	3	∼45	147
GO	MOF–CS	675	MO	Centrifugation + distilled water	∼70	5	>52	127
g-C_3_N_4_	AgI/ZIF-8	455.245	MB	—	94.3	5	90	98
rGO	Zn-MOF	16.47	MB	Deionized water + EtOH	98.6	5	88.1	128
rGO	MIL-125	45	CR	Centrifugation	92.6	4	65.2	96
g-C_3_N_4_	UiO-66(Ce)	709	RhB	Water + EtOH	97.5	5	92.1	130
g-C_3_N_4_/GO	NH_2_-MIL-88B(Fe)	204.86	Direct red 23	Water + EtOH	99	5	92	109
g-C_3_N_4_	Ni/MoS_2_/MOF-2	NA	MB	—	90	5	82	102
g-C_3_N_4_	MIL-53(AI)	43.8	RhB	—	100	5	90	131
rGO	NH_2_-MIL-125(Ti)	750.3	MB	—	100	5	96.7	133
rGO	Fe_2_Ni MIL-88	119	RhB	—	99	5	90.5	148
GO	Nd-MOF	48.80	MB	Water + EtOH	∼95	4	>80	134
GO	ZIF-8	804	Acid red 88	Centrifugation + EtOH/acetone (50 : 50)	85.4	4	∼44	107
g-C_3_N_4_	MOF-5	17.8893	RhB	Double-distilled water	85	3	49	135
GO	ZIF-8/HKUST-1	NA	CR	—	91.81	3	82.91	136
GO	NH_2_-MIL-88B(Fe)	NA	MB	Deionized water	99.63	5	96.06	139
g-C_3_N_4_	MOF-5	308.21	RhB	Washing + calcination	91.5	5	90	110
GO	MIL-100(Fe)	1031	MB	Centrifugation + DMF	>95	10	90	140
g-C_3_N_4_	Ni-MOF	423.2	RhB	—	93	5	∼88	85
g-C_3_N_4_	Ni-MOF	423.2	CR	—	64	5	56	85

a NA = not available.

Typically, the reusability operation involves separating and recovering the spent photocatalyst (GBMOFs in this case) from the reaction mixture after the initial degradation process has come to an end. Subsequently, the sorbed dye and its intermediate byproducts are eluted from the recovered photocatalyst using an eluting/regenerating agent. The eluted or regenerated photocatalyst is then reused in the next round of the dye photocatalytic degradation operation under the same conditions as the initial.^144^ Aside from the number of reuse cycles, the stability of the GBMOF photocatalyst is usually assessed by characterization techniques such as XRD, SEM, and FTIR, to determine whether severe changes to the morphological structure of the GBMOFs have occurred.

**Fig. 12:**
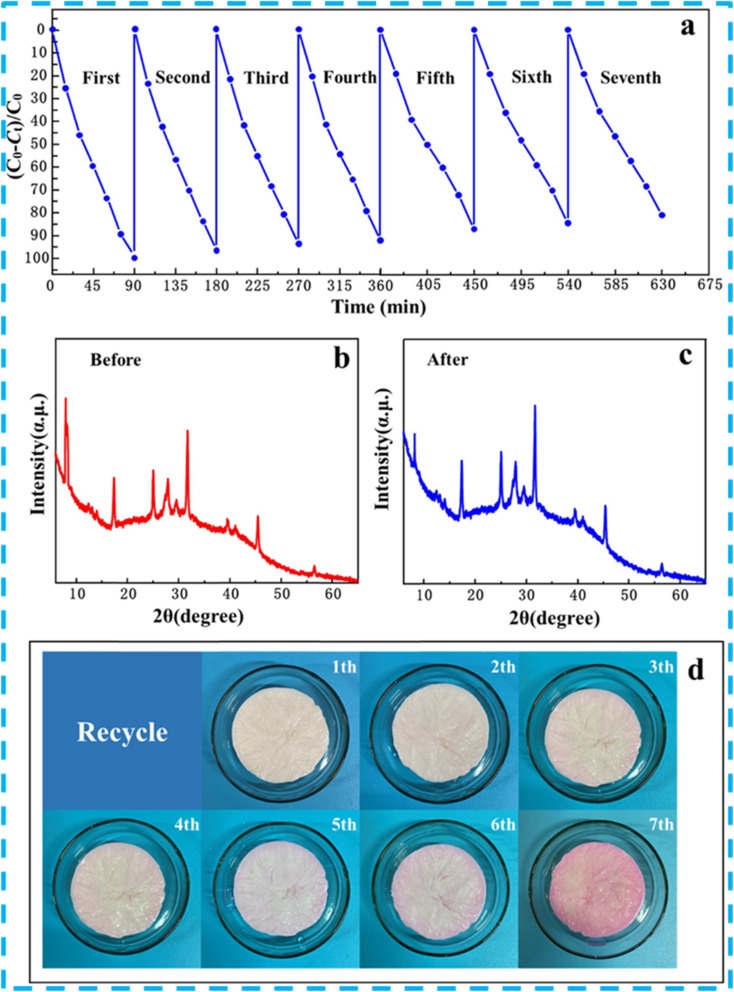
(a) Performance of g-C_3_N_4_/MIL-125/sodium alginates over seven rounds of RhB photocatalytic degradation operation. XRD plots (b) before and (c) after seven reuse cycles. (d) Visual appearance of g-C_3_N_4_/MIL-125/sodium alginate over seven reuse cycles. Reproduced from ref. 126 with permission from Elsevier B.V., J. Zhao, B. Li, Z. Liu, D. Dai, Y. Li, R. Shi and H. Zhang, *Sep. Purif. Technol.*, 2021, **279**, 119696, copyright 2021.

For example, the recyclability potential of g-C_3_N_4_@MIL-101(Fe) for the photocatalytic degradation of RhB was examined over five consecutive cycles. In this study, following the recovery of g-C_3_N_4_@MIL-101(Fe) *via* centrifugation, EtOH was employed as an eluting agent. After that, the photocatalyst material was dried and reused in the next round of the photocatalytic degradation operation. The findings show that g-C_3_N_4_@MIL-101(Fe) exhibits an insignificant loss in its performance after five rounds.^77^ While this suggests the economic potential of the material according to the authors, the lack of a quantitative study on the GBMOF catalyst mass loss after the five reuse cycles undermines the pilot-scale potential. A comparably good photocatalytic degradation efficiency was also reported for the g-C_3_N_4_@MIL-125(Ti) composite,^118^ the g-C_3_N_4_@NH_2_-MIL-53(Fe) aerogel,^116^ and AgI/ZIF-8/g-C_3_N_4_,^98^ for RhB and MB, after four/five recyclability studies, as presented in Table 3. Additionally, it was found that the XRD patterns of pristine and recycled graphene-based MOF are identical, indicating that the recycled material retains its original architectural morphology;^98,116,118^ this is consistent with what was observed for spent g-C_3_N_4_/MIL-125/sodium alginate after being reused seven times for the photocatalytic degradation of RhB, as shown in Fig. 12.^126^ More specifically, as demonstrated in Fig. 12d, the visual colour of the material changes gradually until the 7th round, and this can be attributed to the color change of RhB during degradation; this highlights the fact that GBMOF not only degraded the dye pollutant but also decolourized the dye wastewater. From our own perspective, the strong coordination interactions and hydrogen bonding between the MOF and graphene/g-C_3_N_4_ components, while beneficial for structural stability and recyclability, may also introduce inherent limitations. The enhanced structural rigidity can hinder efficient regeneration, as strongly adsorbed intermediates or degradation by-products may not be easily desorbed, leading to gradual active-site blockage over repeated cycles. This could ultimately compromise catalytic efficiency despite the apparent structural stability. Furthermore, such tightly bound architecture may be more susceptible to irreversible deactivation pathways, including pore blocking and reduced surface reactivity, which are not readily reversed through conventional regeneration methods. From a process perspective, the inability of these materials to lose form may also limit their adaptability in shaping, post-synthetic modification, or integration into scalable systems where some degree of structural flexibility is often required.

In another study,^107^ the recoverability, cyclic stability, and reusability of GO/ZIF-8 for the photocatalytic degradation of acid red 88 were investigated, and four cyclic tests were performed. In contrast to the general trend of washing recovered GBMOF materials with EtOH and water, the GO/ZIF-8 recovered from the reaction mixture by centrifugation was washed with EtOH/acetone (50 : 50). It was observed that as the number of cycles increased, the degradation rates of acid red 88 marginally dropped until they reached around 44% in the last cycle (4th cycle), as compared to >80% from the first cycle to the last cycle in many studies, as presented in Table 3. Logically, this questions the long-term use of GO/ZIF-8 with a mixture of EtOH/acetone as eluent or regenerating agent. According to the authors, the unavoidable loss of GO/ZIF-8 following each centrifugation and washing stage is a possible cause of the decline in photocatalytic efficiency. However, even with a reduction in photocatalytic efficiency, the crystalline morphology of recycled GO/ZIF-8 remains intact when compared to the pristine version.^107^ Nevertheless, it can be opined that the use of a mixture of EtOH/acetone is not strongly effective, because, as observed in Table 3, other studies,^83,109,121^ where similar recovered GBMOF materials were treated with EtOH/H_2_O, delivered >70% dye degradation in the last cycle. From our own perspective, this is due to the fact that EtOH/H_2_O provides a balanced polarity environment that is more effective at desorbing dye molecules and intermediate products. Water enhances the dissolution of ionic/polar dye residues, while EtOH helps solubilize more hydrophobic fragments and reduces surface tension, allowing better penetration into the pores. This synergistic effect promotes more complete cleaning of GBMOF active sites and preserves the coordination environment of the MOF as well as the interfacial interactions with the GO. As a result, the catalyst retains a higher fraction of its active sites, leading to better efficiency even after multiple cycles.

**Fig. 13:**
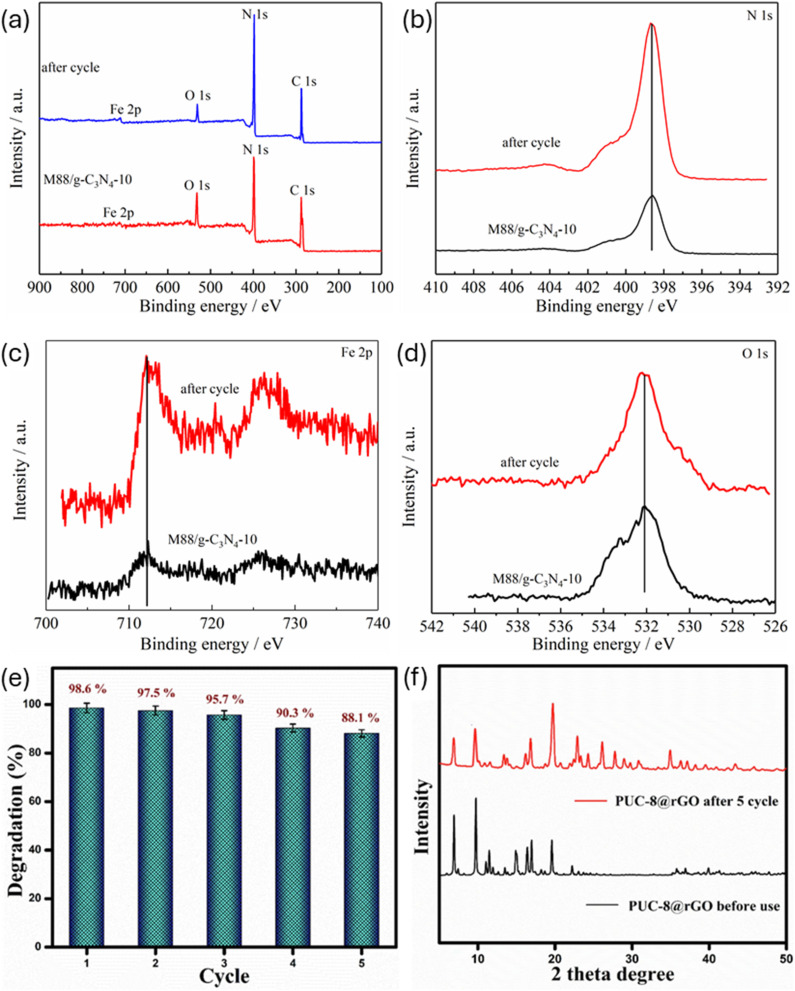
(a–d) XPS spectra of g-C_3_N_4_@M88 before and after 6 recyclability/stability tests for the photocatalytic degradation of RhB. Reproduced from ref. 125 with permission from Elsevier B.V., Z. Shao, D. Zhang, H. Li, C. Su, X. Pu and Y. Geng, *Sep. Purif. Technol.*, 2019, **220**, 16–24, copyright 2019. (e) Recyclability performance and (f) XRD spectra of rGO@Zn-MOF before and after 5 rounds of MB photodegradation. Reproduced from ref. 128 with permission from Elsevier B.V., K. Arya, A. Kumar, A. Sharma, K. Thakur, R. Kumar, S. K. Mehta, S. Singh, V. Kumar and R. Kataria, *Inorg. Chem. Commun.*, 2025, **173**, 113768, copyright 2024.

In another experiment,^125^ the stability and reusability of g-C_3_N_4_@MIL-88A for the degradation of RhB under light irradiation were evaluated, and six-run tests were carried out. Interestingly, after six rounds, the photocatalytic efficiency of the nanocomposite did not exhibit any obvious decrease. Furthermore, the XPS spectra of the material are almost identical before and after the reusability process, as seen in Fig. 13a–d, suggesting that the photocatalyst's structure remains essentially intact. This finding also indicates that neither photodegradation nor recyclability operations affect the functional groups or coordination bonds of g-C_3_N_4_@MIL-88A. Given these results, it can be inferred that MIL-88A/g-C_3_N_4_ has the potential to be employed in actual water remediation.^125^ Similarly, the photostability of Zn-MOF@rGO was investigated by reusing the material five times following recovery from the reaction mixture. In this case study, the recovered spent Zn-MOF@rGO was rinsed thoroughly with water and EtOH and reactivated at 80 °C. As seen in Fig. 13e, following five runs, the material's ability to degrade MB reduced slightly from 98.6% to 88.1%, which can be ascribed to mass loss during the recovery or washing process. Moreover, as presented in Fig. 13f, the XRD analysis of the recycled Zn-MOF@rGO as compared to the pristine version revealed similar patterns, confirming the higher stability and recyclability.^128^ This is consistent with what was documented by Huang and Liu for rGO/NH_2_-MIL-125(Ti), where findings revealed that there was no serious loss of structure or morphological features after achieving over 96% MB degradation efficiency in the 5th round of the recyclability test.^133^ Nevertheless, according to research discoveries, it has been opined that the buildup of small molecules on the GBMOF surface, which may impede the accessibility of light as well as reduce the porosity of the material, may explain the minor decrease in the degradation output over successive cycles.^140^ Additionally, while structural stability is inherently beneficial for multiple recoverability and recyclability efficiency, excessively strong interfacial interactions may reduce the accessibility of catalytic sites or limit mass transfer within the porous structure, particularly if graphene restacking occurs. This could ultimately compromise catalytic efficiency at an industrial reusability level, despite apparent structural stability and recyclability efficiency on the lab scale.

**Fig. 14:**
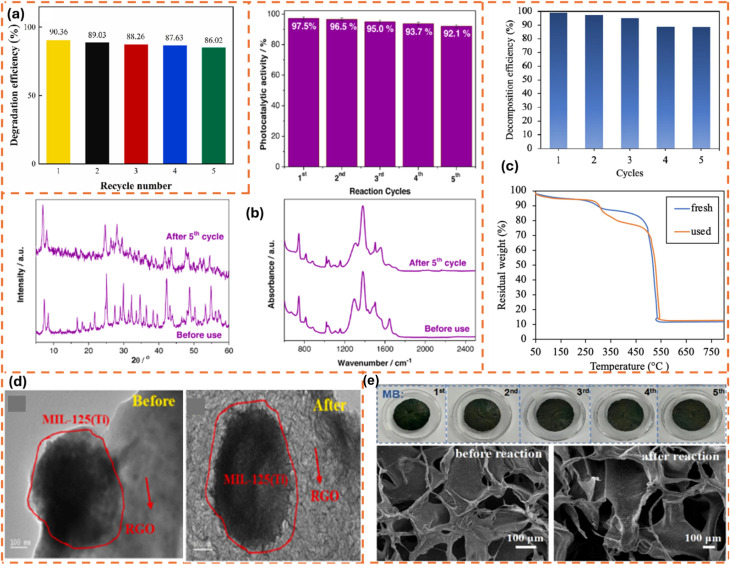
(a) Reusability efficiency of g-C_3_N_4_@MIL-101(Fe) for RhB photodegradation.^69^ Reused with authorization from Elsevier (license code: 6201900224766). (b) Reusability and stability performance of g-C_3_N_4_@CdS@UiO-66(Ce) for RhB photodegradation.^130^ Adapted from Yassin *et al.*, 2025, licensed under CC BY. Open access, which permits unrestricted use, distribution, and reproduction in any medium. (c) Reusability and stability performance of g-C_3_N_4_@MIL-88A for acid red 1 dye photodegradation. Reproduced from ref. 78 with permission from Elsevier B.V., C. E. Tan, E. C. Su and M. Y. Wey, *Appl. Surf. Sci.*, 2022, **590**, 152954, copyright 2022. (d) Stability result of rGO@MIL-125 for CR photodegradation. Reproduced from ref. 96 with permission from Elsevier B.V., R. Fatima, M. N. Afridi, I. Mohdeb, P. Madhusudan and Y. Hwang, *J. Water Process Eng.*, 2025, **69**, 106893, copyright 2024. (e) Recyclability and stability results of GO@NH_2_-MIL-88B(Fe) for MB photodegradation. Reproduced from ref. 139 with permission from Springer Nature, X. Lin, J. Zhao, Y. Zhang, Y. Li, Y. Liao and H. Zhang, *J. Polym. Environ.*, 2024, **32**, 2091–2104, copyright 2023.

Similarly, after five successive recyclability tests of rGO@MIL-125 for the photocatalytic degradation of CR, the performance remained strong. According to findings, the aggregation and deposition of pollutants on the rGO@MIL-125 surface, as well as the interaction of CR with the material's molecules, may be responsible for the slight decrease in degradation output. Furthermore, as shown in Fig. 14d, the study revealed no serious structural changes in rGO@MIL-125 after the five reuse rounds, suggesting that the material is stable and eco-friendly for real-world uses.^96^ In another case study,^130^ the stability and recyclability of g-C_3_N_4_@CdS@UiO-66(Ce) for the degradation of RhB were examined. In this experiment, the spent GBMOF material was cleaned three times with water, twice with EtOH, and dried at 60 °C to elute any residual RhB dye before subsequent use. Notably, following five consecutive reuses, a high RhB degradation efficiency was still attained, as shown in Fig. 14b. Amazingly, even after five rounds of reuse, the g-C_3_N_4_@CdS@UiO-66(Ce) structure did not change, according to the XRD and FTIR study presented in Fig. 14b. These findings demonstrate the high stability and good recyclability of g-C_3_N_4_@CdS@UiO-66(Ce) as well as its potential for industrial-scale application.^130^ Nonetheless, the claim of eco-friendliness, stability, and industrial applicability needs further evidence through studies on possible mass loss and leaching of metals like Cd, Ce, and Fe. For instance, Huang *et al.*^94^ observed that GO/MIL-101(Fe)/PANCMA still delivered over 73% RhB degradation efficiency even after 30 reuse cycles without any significant loss in the structural coordination and morphology of the material.^94^ This result is consistent with reusability/stability findings for various GBMOFs from other studies, shown in Fig. 14a, and c–e.^69,78,96,139^

Notably, the findings revealed a consistent pattern of good recoverability and reusability of GBMOFs with a slight to moderate reduction in the dye photocatalytic degradation efficiency in the last cycle. However, from our critical perspective, several important research gaps and limitations emerge from the current body of work.

The first critical limitation noticed is the lack of standardized protocols for evaluating catalyst recyclability, which significantly undermines cross-study comparability. Regeneration strategies vary widely, ranging from centrifugation and filtration to ethanol washing, water rinsing, and calcination, yet the underlying regeneration mechanisms are rarely investigated. Also, a consistent decline in performance is observed upon reuse and, in some cases, drops below 80%. From our own perspective, this clearly indicates progressive GBMOF catalyst deactivation rather than just efficiency reduction, as concluded in many studies. Potential causes of deactivation, such as photocorrosion of MOF nodes, pore blockage by dye intermediates, graphene restacking, and metal leaching, that can occur during multiple reuses, remain underexplored. This is a crucial research gap that needs to be filled in future work. Moreover, there is a notable absence of quantitative stability metrics, as most studies report only percentage degradation without accounting for catalyst mass loss or metal ion release during recovery or recycling. This study gap is not trivial, as it raises concerns regarding the risk of secondary environmental contamination and restricts the environmental relevance of current findings. Another important issue observed is the lack of kinetic studies throughout the recyclability experiments. Although percentage degradation efficiencies are routinely presented, the evolution of reaction kinetics across cycles, such as changes in rate constant or reactive species generation, is rarely monitored, limiting a deeper understanding of GBMOF catalyst stability.

Also, the limited inclusion of structurally complex pollutants, such as mixed dye contaminant systems or real wastewater systems, highlights a disconnect between laboratory evaluations and practical application scenarios within the context of recoverability, regenerability, stability, and reusability. Given the presence of competing ions, natural organic matter, and fluctuating physicochemical conditions in real wastewater systems, the reported performances under simplified conditions observed across studies may be overly optimistic. Furthermore, most regeneration and recovery approaches are inherently laboratory-scale, with heavy dependence on centrifugation and similar batch techniques that are impractical for industrial deployment. The lack of emphasis on scalable strategies such as continuous-flow operation, catalyst immobilization, or magnetic separation further constrains industrial applicability potential. All of these identified research gaps need to be addressed by future researchers to advance the GBMOF photocatalytic techniques and compete with existing wastewater remediation methods.

## 8. Practical applicability and pilot scale potential

Practical applicability and pilot-scale potential of any material or method point to its industrial relevance and technology readiness level.^149,150^ Yet, there is a dearth of studies in this direction within the context of GBMOFs, as most work remains at the laboratory scale. However, based on the limited amount of available literature, in the next subsections, the practical applicability is succinctly discussed and evaluated in relation to GBMOF performance in real wastewater systems, metal ion leaching potential, and economic feasibility on a large scale.

### 8.1 Performance in real wastewater and multi-pollutant systems

In practice, wastewater usually contains a combination of pollutants rather than a single dye pollutant, as co-existing pollutants often occur simultaneously, making treatment strategies more challenging.^151,152^ Moreover, surface water, rivers, groundwater, and industrial effluent typically contain varied concentrations of heavy metals, pharmaceuticals, dyes, and other harmful contaminants that pose significant risks to human health and the environment.^151^ Thus, to evaluate the practical applicability of GBMOFs, it is crucial to conduct either direct field trials or laboratory remediation experiments that mimic real-world conditions.^152^ Unfortunately, this aspect of research is often overlooked by researchers, leading to a shortage of studies in this area. Nevertheless, within the few available articles, the potential of GBMOFs for the photocatalytic degradation of dye pollutants in real/mimic wastewater is discussed.

**Fig. 15:**
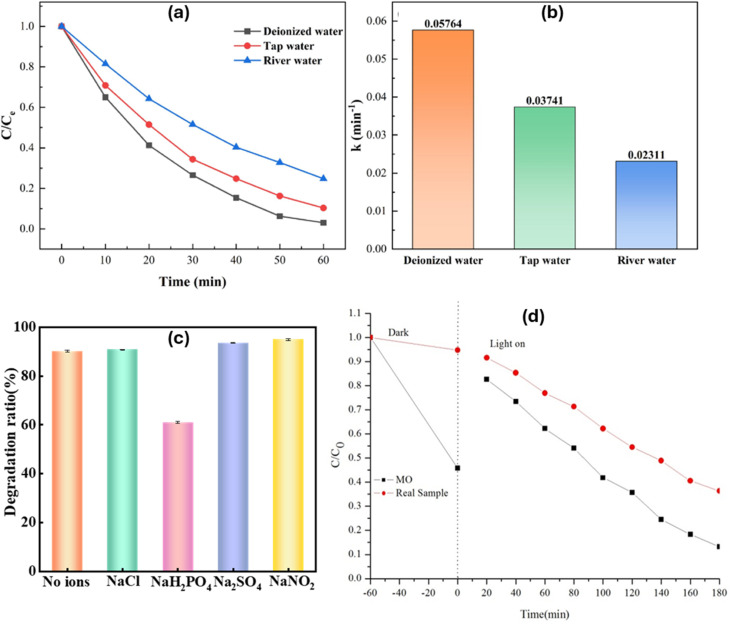
(a) Photocatalytic degradation efficiencies and (b) reaction rates of UiO-66/g-C_3_N_4_ for RhB in different tap water, deionized water, and river water. Reproduced from ref. 72 with permission from Elsevier B.V., D. Lan, H. Zhu, J. Zhang, C. Xie, F. Wang, Y. Zheng, Z. Guo, M. Xu and T. Wu, *Appl. Surf. Sci.*, 2024, **655**, 159623, copyright 2024. (c) Influence of co-existing ions on the photocatalytic degradation of methylene blue by SrAl_2_O_4_:Eu^2+^,Dy^3+^/g-C_3_N_4_@NH_2_-UiO-66. Reproduced from ref. 154 with permission from Elsevier B.V., M.-L. Chen, S.-S. Li, L. Wen, Z. Xu, H.-H. Li, L. Ding and Y.-H. Cheng, *J. Colloid Interface Sci.*, 2023, **629**, 409–421, copyright 2022. (d) Photocatalytic degradation of MO and real textile wastewater using UiO-66(Zr)/rGO/Ag_3_PO_4_. Reproduced from ref. 153 with permission from Elsevier B.V., A. Hadush, T. Kebede, A. M. Taddesse, N. R. Habib and M. Sánchez-Sánchez, *Opt. Mater.*, 2024, **155**, 115901, copyright 2026.

For example, in a particular investigation,^153^ the practical applicability of UiO-66(Zr)/rGO/Ag_3_PO_4_ was evaluated using a real textile effluent collected from Bahirdar Textile Share Company, Ethiopia. In this study, the untreated effluent was directly subjected to photocatalytic degradation under optimized conditions. As shown in Fig. 15d, the degradation efficiency for the model dye pollutant (methyl orange) reached 89.8%, whereas a lower efficiency of 63.6% was observed for the real wastewater sample. The reduced performance in the real effluent was attributed to the complex composition of the wastewater, which contains a mixture of dyes and inorganic ions that can compete for active sites on the catalyst surface. In addition, the nature and charge of the dye species play crucial roles, as the GBMOF catalyst surface (pH_pzc_ ∼6.1) may preferentially interact with specific dye types depending on their ionic character. Furthermore, the higher concentration and diversity of contaminants in the real sample can hinder light penetration, thereby limiting the generation of photogenerated charge carriers (e^−^/h^+^). As a result, fewer active species are available for degradation, leading to lower overall photocatalytic efficiency compared with the model system.^153^ Thus, a key limitation is the significant performance drop in real wastewater due to matrix complexity, coupled with a lack of systematic strategies to mitigate interference from co-existing ions, mixed pollutants, and light attenuation effects. In a comparable study, the practical applicability of UiO-66/g-C_3_N_4_ photocatalyst was assessed in different water matrices, including deionized water, tap water, and river water, to simulate realistic environmental conditions. As presented in Fig. 15a, the degradation efficiency reached 97.96% within 60 min in deionized water, whereas lower efficiencies were recorded for tap water (89.65%) and river water (75.21%) under the same conditions. However, with prolonged reaction time, the systems in tap and river water were able to approach similar removal performance. Kinetic analysis (Fig. 15b) further revealed that the reaction rates in tap water (0.03741 min^−1^) and river water (0.02311 min^−1^) were significantly lower than that in deionized water, confirming the inhibitory effect of real water constituents. This decline in performance was mainly attributed to the presence of organic matter and inorganic ions, which can compete with target pollutants for ROS and active sites. In particular, ions such as Cl^−^ may react with photogenerated holes h^+^ and ˙OH, thereby reducing the availability of these reactive species.^72^

In another study,^154^ the influence of coexisting anions on the photocatalytic degradation of methylene blue using SrAl_2_O_4_:Eu^2+^,Dy^3+^/g-C_3_N_4_@NH_2_-UiO-66 was systematically investigated at pH 6. Different anions, including NaH_2_PO_4_, NaCl, Na_2_SO_4_, and NaNO_2_ (0.05 mol L^−1^), were introduced into the reaction system. The results indicated that most of the tested anions had no significant inhibitory effect on MB degradation, except for NaH_2_PO_4,_ as shown in Fig. 15c. Interestingly, the presence of nitrite and sulfate ions slightly enhanced the photocatalytic performance. This improvement was attributed to their ability to interact with photogenerated h^+^, leading to the formation of additional oxidative species, while also promoting the generation of ˙OH through reactions with H_2_O molecules. Consequently, the presence of these anions increased the concentration of reactive species and improved the separation efficiency of e^−^/h^+^, likely due to the electrostatic field formed at the UiO photocatalyst surface.^154^ In another mimicked real dye wastewater, when potassium iodide was introduced into the photocatalytic reactor, the degradation performance of g-C_3_N_4_@UiO-66 for MB slightly decreased. This is because KI scavenges h^+^, which also plays some roles in the photocatalytic degradation of MB directly or indirectly.^70^ This is comparable to the moderate negative effect of NaI and Cr(vi) on the performance of rGO/Zn-MOF for the photocatalytic degradation of MB^128^ and g-C_3_N_4_@MIL-53(Al) for RhB.^131^ Moreover, from our own perspective, the Cr(vi) and individual ions present in KI and NaI can act as co-existing ions to either compete for active photocatalyst sites, hinder light, or reduce radical attack activity.

Also, in another study on the real-time commercial application of GBMOF, NiCu@INA/rGO was employed for the photocatalytic treatment of mixed dye, as such conditions closely resemble real industrial effluents containing multiple coexisting pollutants.^83^ In this context, the NiCu@INA/rGO GBMOF demonstrated strong performance for simultaneous dye degradation. RhB degradation remained consistently high (∼97%) across all dye mixture ratios, whereas OrG degradation was composition-dependent, reaching a maximum of ∼92% at a 1 : 1 ratio. The lower efficiency of OrG suggests competitive interactions, where RhB preferentially occupies active sites and suppresses OrG degradation. This behavior can be attributed to the fact that RhB exhibits better interaction with GBMOF. In contrast, the anionic nature of OrG results in weaker surface interactions and a greater reliance on hole-mediated oxidation, leading to reduced removal efficiency. Additionally, NiCu@INA/rGO GBMOF displayed good recoverability and reusability for five cycles, with only 5.2–10% loss of activity, which is ascribed to loss of material during the recycling process, and slight blockages of activity due to diffused dye molecules in the pores of the photocatalyst.^83^ From our own perspective, the GBMOF catalyst shows promising potential for stable, real-world wastewater treatment involving complex pollutant mixtures. However, a key gap lies in addressing competitive adsorption and selectivity limitations, particularly for anionic species, which remains critical for optimizing performance in realistic water matrices. Moreover, reporting bare material loss without reporting the actual mass loss or exploring leaching potential after multiple reuse cycles in a mimicked real dye wastewater treatment is a critical study gap that needs to be addressed in future works. A study by Arya *et al.*^128^ on photocatalytic degradation of MB revealed that the rGO/Zn-MOF employed could pose cytotoxicity risks to biological systems if leached, and this reinforces the need for careful evaluation of material leaching behavior.

### 8.2 Metal ion leaching potential

Another important factor essential for evaluating the real-world applicability of GBMOF photocatalysts is the potential for metal ion leaching from the MOF component. This study is crucial because if the metal ion leaching is beyond the acceptable limit, it could defeat the original intention of environmental remediation. Unfortunately, only two studies are available regarding GBMOF for dye photocatalytic degradation. In one of these studies,^127^ to investigate the leaching of Cu ions from the surface of Cu-MOF–CS/GO, the material composition was characterized using ICP before and after the MO photocatalytic degradation operation. Findings from the study revealed that there was only a slight change in the percentage of Cu metal ion present in the Cu-MOF–CS/GO GBMOF after the degradation process (10.69%) compared to before (11.57%). Interestingly, the percentage composition of oxygen, hydrogen, and nitrogen increased slightly.^127^ While the authors did not give any tangible explanation for such an increase, it is reasonable to attribute this to sorption of the parent dye molecules and degradation by-products on the GBMOF surface. This confirms the efficiency and stability of the Cu-MOF–CS/GO photocatalyst. Nevertheless, this does not totally establish the practical or industrial applicability because the little observable Cu that leached (7.61%) was not measured in mg L^−1^ to determine whether it was beyond the WHO acceptable limit for drinking/clean water. This gap should be addressed in future studies.

Similarly, in another leaching study, Bai *et al.*^134^ investigated the effect of pH on the release of Fe ions from GO@Fe_3_O_4_@Nd-MOF following photocatalytic degradation of MB. The ICP analysis shows that 2.166 and 0.221 mg per L Fe ions leached at pH 3 and 5, respectively, which is equivalent to ∼1.6% and 0.2% of 133 mg per L GO@Fe_3_O_4_@Nd-MOF.^134^ The amount of Fe leached at pH 3 is beyond the typical acceptable limit (<0.3 mg L^−1^), above which the treated water could be brown, give a bad metallic taste, and become unfit for public use. This raises a concern about the practical applicability of GO@Fe_3_O_4_@Nd-MOF for real effluent from acid mine drainage and the mining industry, *etc.*

### 8.3 Techno-economic analysis and economic feasibility

Notably, harmonizing material innovation with economic feasibility cannot be overlooked in any sustainable solution. The techno-economic analysis is crucial because it is undeniably a vital tool in decision-making and one of the important areas of interest or consideration for investors, industries, engineers, lawmakers, and other key players.^56,155,156^ This is especially true when selecting an eco-benign and cost-effective treatment technique to remove pollutants such as dyes.^157^ Furthermore, techno-economic analysis is essential for promoting a certain method or material and determining its sustainability in the industry.^157,158^ Generally, the cost of the treatment technique is usually heavily dependent on the cost of the material employed for the remediation process.^155^ More specifically, material costs are key to determining the financial implications of large/field-scale industrial operations.^159^

Unfortunately, there are no sufficient studies on the techno-economic analysis of GBMOFs for dye degradation. However, in a particular study on GO@Fe-MOF and GO@Al-MOF for MO and MB dye remediation, a simple technoeconomic analysis was given on the cost of the two GBMOF materials.^159^ According to the authors' analysis, it costs $0.39 and $0.15, respectively, to produce 1 g of GO@Al-MOF and GO@Fe-MOF from industrial-grade chemicals. From this short analysis, it was estimated that the costs of GO@Al-MOF and GO@Fe-MOF to clean up 1 g of dye pollutant are $0.44–0.68 and $0.39–0.62, respectively. From our own perspective, in comparison to conventional materials such as TiO_2_, which costs approximately $1.50–2.00 per kg,^160^ and commercial activated carbon, which can cost around $1.5–10 per kg,^161,162^ GBMOFs are not presently cost-effective. However, there is still a need for more serious studies on the techno-economic analysis to substantiate this, while future researchers continue to work on synthesizing very cheap GBMOFs that can compete economically with existing photocatalyst materials.

## 9. Key emerging trends in GBMOFs for the photocatalytic degradation of dyes

Recent advances in GBMOF photocatalysts demonstrate a clear transition from conventional single-component MOFs or GO toward highly integrated, multifunctional, heterostructured systems tailored for efficient pollutant degradation. Notably, early investigations usually explored pristine MOFs or bare g-C_3_N_4_ and graphene-based materials. However, their full-fledged photocatalytic performance was often limited by wide band gaps, rapid charge recombination, and insufficient visible-light utilization. As a result, current research is increasingly focusing on interfacial engineering through the rational integration of conductive graphene derivatives and visible-light-active g-C_3_N_4_ with MOFs to construct efficient heterojunction composites. This can allow light harvesting, charge separation, and dye adsorption to be boosted concurrently by leveraging the high surface area as well as tunable porosity of MOFs alongside the excellent electron mobility of graphene and the suitable band structure of g-C_3_N_4_.

**Fig. 16:**
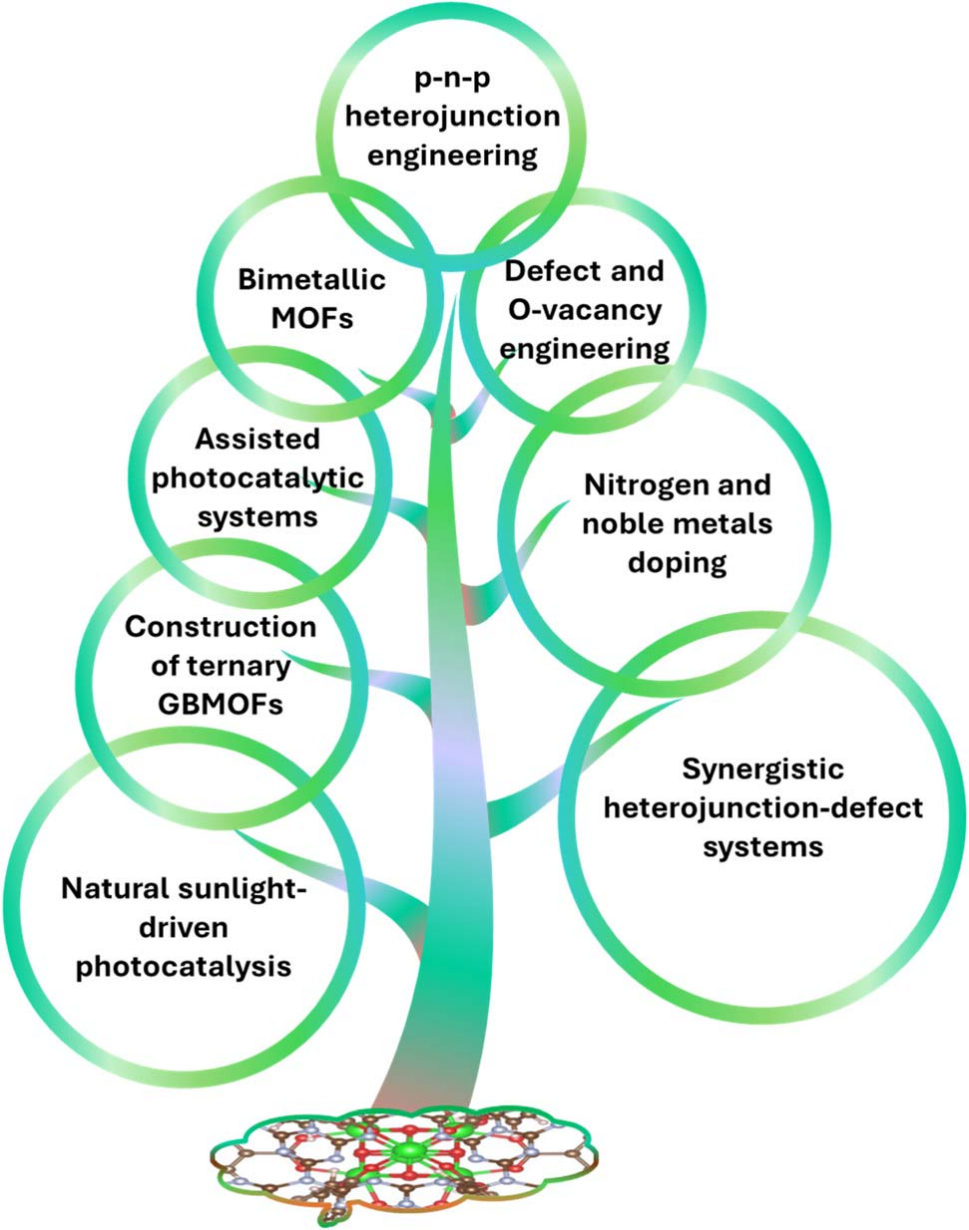
Key emerging trends in graphene-/graphitic carbon nitride-based metal–organic frameworks for photocatalytic dye wastewater treatment.

As displayed in Fig. 16, one of the key emerging trends is the construction of advanced GBMOF photocatalysts with p–n and p–p–n heterojunction architectures. Typically, GBMOFs behave as an n–n type and Z-/S-scheme semiconductor composite. Specifically, when g-C_3_N_4_ or rGO is integrated with n-type MOFs, such as Zr, Fe, Ti, or Zn-based frameworks, n–n heterojunctions are commonly formed.^78,88,101,125^ Then, interfacial band alignment and Fermi-level equilibration induce directional charge migration, generating an internal electric field that promotes efficient e^−^/h^+^ separation. In many GBMOFs, this charge-transfer pathway conforms to a Z-/S-scheme heterojunction, wherein photogenerated electrons with weak reduction ability and holes with weak oxidation ability recombine at the interface, preserving highly reactive charge carriers for photocatalytic reactions. The introduction of graphene, GO, or rGO further accelerates electron transport and suppresses interfacial recombination by acting as a conductive electron tank. These n–n Z-/S-scheme heterostructures synergistically combine visible-light responsiveness, high surface area, and efficient charge separation, resulting in improved dye degradation performance while maintaining structural simplicity and long-term aqueous stability, making them promising for scalable wastewater treatment applications.^78,83,88,125^

Interestingly, in recent times, photocatalytic enhancement in GBMOF systems is increasingly achieved through p–n–p heterojunction architectures, which have emerged as highly efficient charge-regulation frameworks. In such systems, an n-type MOF is sandwiched between two p-type components, as seen in GO@MOF@CuO for MB degradation, where p-type CuO and GO have their Fermi levels positioned adjacent to their VB edges, and the MOF has its Fermi level positioned adjacent to its CB.^104^ The asymmetric charge diffusion across the dual p–n interfaces leads to the formation of extended depletion regions and a strong built-in electric field within the GBMOF composite. This internal electric field effectively drives photogenerated e^−^/h^+^ toward spatially distinct domains under light exposure, significantly inhibiting charge recombination. As a result, p–n–p heterojunctions exhibit enhanced charge-carrier separation efficiency, prolonged carrier lifetimes, and elevated ROS generation. These synergistic effects translate into accelerated and more effective dye degradation, highlighting p–n–p architecture as a powerful design strategy for next-generation GBMOF photocatalysts.

Another emerging field is in the area of defect engineering, which has arisen as an effective strategy to tailor the electronic structure of GBMOFs by narrowing the band gap, boosting visible-light striking/absorption, and promoting ROS generation during photocatalysis.^101^ In these systems, defects such as nitrogen vacancies in g-C_3_N_4_ and oxygen defects in graphene derivatives act as electron-trapping sites, thereby prolonging charge-carrier lifetimes and facilitating surface redox reactions with adsorbed dye molecules.^101,163^ When defect-rich components are integrated into heterojunction architectures, such as defect-engineered GO@Ag–Zn-BTC,^101^ synergistic enhancements in adsorption capacity, light harvesting, interfacial charge transfer, and photocatalytic kinetics are achieved. This combined effect between defect states and heterojunction-induced internal electric fields significantly boosts photocatalytic activity and underscores defect engineering as an emerging powerful tool for high-performance GBMOF-dye degradation systems.

From an environmental sustainability perspective, the use of natural sunlight represents another emerging direction in GBMOF photocatalysis operation.^85,103,110^ This is gaining popularity because it saves energy usage, time, and costs. In addition to the use of natural sunlight, another noteworthy emerging trend in GBMOF dye degradation research is the development of assisted photocatalytic systems, particularly those coupled with oxidants such as H_2_O_2_,^108,109,122^ as well as ultrasound-assisted processes.^107^ These hybrid AOP systems boost dye degradation remarkably by aiding GBMOFs to generate more ROS compared to the photocatalytic degradation process alone. The synergistic integration of photocatalysis and other advanced oxidation processes is increasingly recognized as an effective strategy for enhancing the degradation of stubborn dye pollutants.

As observed in literature, another developing research focus is the integration of dopants and co-catalysts such as noble metals (*e.g.*, Ag) and non-metals (*e.g.*, nitrogen). Doped GBMOF versions, such as g-C_3_N_4_@Ag_3_PO_4_/Zr-BDC,^75^ Ag@rGO@MIL-125(Ti),^99^ N-doped g-C_3_N_4_@NH_2_-MIL-125(Ti),^88^ and N-doped ZIF-8@rGO,^97^ exhibit narrowed band gaps and increased surface defects, thus advancing visible-light activity and higher quantum yields. Similar to this is the emerging use of bimetallic MOFs for novel GBMOF fabrication, such as GO@Cu/Cd bimetallic MOF^100^ and rGO@NiCu-MOF.^83^

A new frontier has also been opened up in the area of ternary GBMOF composites engineering, which combines binary GBMOF with other materials such as metal oxide nanoparticles for dye photocatalytic degradation. These ternary systems leverage the synergistic properties of each component to enhance the inherent properties of GBMOFs. By constructing heterostructured interfaces among all three components, the overall photocatalytic performance of GBMOF is increased by improving surface-active site accessibility and reactive oxygen species generation, among others. Overall, ternary GBMOFs, such as g-C_3_N_4_@ZnFe_2_O_4_@UiO-66,^113^ GO@BiOBr@MOF-5,^119^ GO@MOF@Bi_2_O_3_,^124^ gC_3_N_4_/Fe_3_O_4_@Zn-MOF-5,^135^ GO@MOF@CuO,^104^ and GO@MIL-53(Fe)@BiVO_4_,^117^ have demonstrated excellent dye degradation efficiencies compared to their binary counterparts, underscoring their potential scalability for real wastewater treatment. Nevertheless, despite the improved synergistic properties and photocatalytic efficiency of ternary GBMOFs, uncertainty remains as to whether their synthesis cost is proportional to their enhanced dye degradation performance relative to binary GBMOFs.

## 10. Technology readiness level

**Fig. 17:**
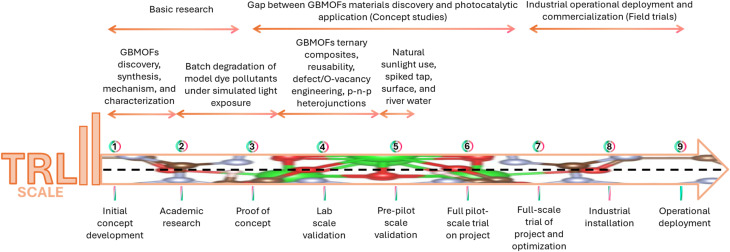
Technology readiness level of the GBMOF-photocatalytic systems.

Technology readiness level (TRL) is a scoring scale (usually from 1–9) employed to assess or judge the maturity status of a process, from original concept to full operational implementation. As illustrated in Fig. 17, TRL 1 and 2 characterizes understanding elementary concepts and principles, TRL 3 and 4 deals with lab-scale proof-of-concept/functionality, TRL 5 and 6 represents process development and system integration to pre-pilot scale, TRL 7 and 8 covers optimization and pre-commercialization, while TRL 8 and 9 involves large-scale commercial operation.^164,165^ Interestingly, as shown in Fig. 17, since the evolution of GBMOF photocatalytic systems for dye pollutants degradation, it is presently positioned at early TRL stages (TRL 1–4), with little advancement toward system-level validation, despite the major performance trends, advancements, and emerging trends recorded already as covered in the preceding sections. While photocatalysis, as an AOP and innovative water treatment technique,^166,167^ has attained higher TRL levels for certain materials, such as MOFs,^167^ rGO,^168^ and TiO_2_,^169^ GBMOF systems are still largely restricted to laboratory-scale demonstrations.^170,171^

Specifically, at TRL 1 and 2, research efforts are mainly focused on elementary comparative investigations of bare MOFs, GO/rGO, and g-C_3_N_4_ against GBMOFs. These studies focus on basic construction, band gap narrowing, defect chemistry, oxygen vacancy formation, surface electronic states, and charge-carrier generation and transport. Photocatalytic degradation of dyes at this stage typically serves as proof-of-concept, carried out under UV or simulated solar irradiation, such as Xe or fluorescent lamps, using model dye aqueous solutions.^131,133^ Therefore, rather than proving technological readiness, TRL 1 and 2 research is primarily focused on GBMOF synthesis, characterization, reaction processes, and comparative studies.

Advancement to TRL 3 and 4 has become increasingly apparent with the development of advanced engineered GBMOFs. These include heterojunction architectures such as p–n–p and ternary composites with narrower band gap, Z-/S-scheme systems, defect and oxygen vacancy, use of bimetallic MOFs, nitrogen and noble metal doping, and the use of GQD.^83,97,104,120^ At this stage, photocatalytic degradation performance is usually validated through controlled batch experiments in the lab using representative dye pollutants such as MB, MO, and RhB. Performance evaluation, usually involving kinetic analyses, radical scavenger studies, and short-term stability/reusability testing (commonly 3–30 cycles),^75,94^ is used to achieve laboratory-scale validation.

Notably, a small but growing number of studies have progressed to assess the photocatalytic degradation performance of GBMOFs in spiked real-water matrices such as tap water, industrial effluent, surface water, and river water.^72,153^ In these studies, dye pollutants were deliberately introduced into natural water samples to evaluate photocatalytic degradation under the influence of background ions and natural organic matter. The successful degradation of dye (75.21% efficient) using GBMOFs like g-C_3_N_4_@UiO-66 in such mimic complex aqueous environments (river water) represents an important step toward environmental relevance as it partially bridges the gap between bare laboratory systems and ideal wastewater conditions.^72^ Even though these studies remain scant, are batch-based, and do not yet constitute or establish full system validation, they may reasonably be positioned at the upper part of TRL 4, as they demonstrate functional performance with non-ideal water matrices.

Despite these advances, TRL 5 and above remain largely unexplored for the GBMOF-based dye degradation method. Only isolated reports have investigated operation under natural sunlight, or treatment of mixed-dye systems that are more closely true to real dye wastewater.^72,85,110^ Importantly, the shift from laboratory validation (TRL 4) to pilot-scale implementation (TRL 5 and 6) requires demonstrations in continuous-flow photoreactors, long-term operational stability (for weeks and months rather than minutes hour^−1^), leaching assessments, and validation under very realistic hydraulic and optical conditions.^172^ These requirements have not yet been comprehensively addressed for GBMOF photocatalytic systems.

Additionally, TRL progression is naturally non-linear and non-cumulative, in that fulfilling the conditions of one level does not automatically translate into advancement to the next.^166^ The absence of standardized testing protocols, comparative benchmarking, and systems-level optimization further complicates TRL advancement and limits cross-study reproducibility. As a result, while GBMOF material fabrication and application have attained some sort of scientific maturity level, the lack of integrated reactor design and scalability studies has constrained the technology from advancing beyond early-stage readiness.

Overall, the current TRL situation shows a distinct dichotomy between the outstanding performance of GBMOF photocatalysts in laboratories and their low level of technological preparedness for treating dye wastewater in real-world settings. A strategic change from material-focused innovation to real application-oriented validation, with an emphasis on reactor integration, operational robustness, and scalability under actual conditions, will be necessary for any meaningful advancement from the present TRL 1–4 toward higher TRL levels.

## 11. Future perspectives

GBMOFs represent a rapidly evolving class of functional photocatalysts with significant potential for environmental remediation. The future of GBMOFs in photocatalysis lies in the synergistic integration of interfacial engineering, advanced heterojunction design, and scalable material processing, supported by fundamental mechanistic insights. These efforts will be pivotal in translating GBMOF photocatalysts from academic research to real-world sustainable technologies. Some key future research directions can be identified.

### 11.1 Perspective for full-scale wastewater treatment

**Fig. 18:**
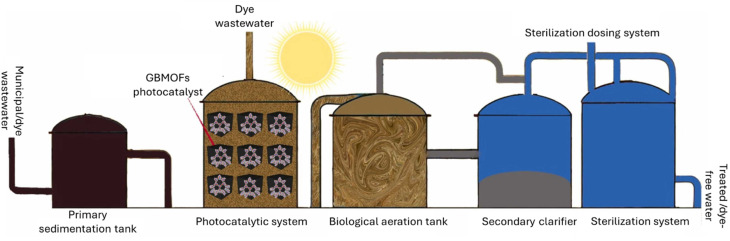
Potential integration of the GBMOF photocatalytic degradation systems with existing wastewater treatment infrastructures. Adapted from ref. 173 with permission from Elsevier B.V., M. Ahmad and M. Yousaf, *J. Environ. Chem. Eng.*, 2025, **13**, 118374, copyright 2025.

Future translation of GBMOF photocatalysts from laboratory studies to full-scale wastewater treatment will depend on their seamless integration into existing wastewater treatment infrastructures, as shown in Fig. 18, and the development of scalable photoreactor systems. The modular nature of GBMOFs enables their incorporation into conventional unit operations, such as slurry reactors, immobilized catalyst beds, membrane-assisted systems, and hybrid advanced oxidation processes, thereby complementing biological and physicochemical treatment stages. Reactor design considerations, including uniform light distribution, effective mass transfer, catalyst recovery, and thermal management, will be critical to maintaining high photocatalytic efficiency under continuous-flow conditions. The ability of GBMOFs to operate efficiently under natural sunlight offers a significant advantage by reducing energy input and operational costs, positioning this technology as a viable option for energy-efficient treatment of dye-laden effluents from textile, pharmaceutical, and dye-manufacturing industries. Continued pilot-scale demonstrations and life-cycle assessments will be essential to validate their long-term stability, economic feasibility, and environmental sustainability in real wastewater matrices.

### 11.2 Rational interface and band structure engineering

Future advances will rely on precise control over GBMOFs interfacial chemistry to tailor band alignment and charge-transfer pathways.^174^ Strategies such as linker functionalization, metal-node substitution, and graphene doping are expected to enable the rational construction of type II, Z-scheme, and S-scheme heterojunctions that simultaneously maximize charge separation and preserve strong redox potentials. Computational screening combined with *in situ* spectroscopic techniques will play an essential role in guiding this interface design.^175^

### 11.3 Enhanced light harvesting and solar utilization

Expanding the optical response of GBMOFs into visible and near-infrared regions remains a critical challenge. Bandgap engineering through ligand modification, defect introduction, and plasmonic coupling with graphene is anticipated to significantly improve solar energy utilization. Hybridization with photosensitizers or co-catalysts may further enhance photon absorption and charge injections.

### 11.4 Mechanistic understanding and *in situ* characterization

A deeper understanding of charge carrier dynamics, ROS generation, and interfacial reaction kinetics is essential for rational catalyst optimization.^176,177^ Advanced *in situ* techniques, such as transient absorption spectroscopy, electron paramagnetic resonance, and synchrotron-based methods, will be crucial for elucidating structure–activity relationships at the atomic and molecular levels.

## 12. Conclusion

This review provides a critical and comprehensive analysis of recent advances in GBMOFs for the photocatalytic degradation of various hazardous dye pollutants. Several key findings and takeaways were derived from the study. Findings show that a good number of GBMOF photocatalytic systems can accomplish quite excellent degradation efficiency within 3–390 minutes at pH 3–13, light intensity of 16–500 W, and dose of 2 to ∼1000 mg. This remarkable degradation efficiency was observed to be largely facilitated by ˙OH and ˙O_2_^−^, and h^+^ in a few cases. Nevertheless, one critical insight derived from this review is that high degradation efficiency does not always translate to complete mineralization of the dye pollutant, high quantum efficiency, or excellent TOC removal. Furthermore, the study shows that GBMOFs can be recycled 3–30 times while maintaining >70% of their original degradation performance and morphology structure. Although problematic issues, such as metal ion leaching potential, have not been adequately addressed in studies, the excellent performance of GBMOFs can be attributed to the synergetic properties, such as improved light absorption, narrower band gaps (as low as 1.75 eV), heterojunction architecture, reduced e^−^/h^+^ recombination, enhanced charge transfer and electron mobility, and the availability of more photocatalytically active sites. Recent advances and emerging trends include the application of p–n–p heterojunctions, the use of natural sunlight, an assisted photocatalytic degradation system, oxygen vacancies, defect engineering, and ternary GBMOFs. Moreover, it was discovered that ternary GBMOFs outperform their binary GBMOF counterparts and individual components, while the assisted photocatalytic system outshines non-assisted ones. Also, natural sunlight can compete tightly with common irradiation sources such as xenon, mercury, and fluorescent lamps. This suggests the industrial-scale potential of the GBMOF-based photocatalytic degradation method. Finally, future perspectives were presented in order to establish a clear framework for guiding the design of highly efficient, stable, practical, and enhanced GBMOF photocatalytic systems for the remediation of various dyes and other pollutants.

## Author contributions

Stephen Sunday Emmanuel: conceptualization, methodology, writing – original draft preparation, date curation, formal analysis, validation, writing – reviewing and editing, supervision, project administration, Gloria Onome Achurefe: writing – original draft preparation, validation, writing – reviewing and editing, Sefiu Olaitan Amusat: writing – original draft preparation, validation, writing – reviewing and editing, Ademidun Adeola Adesibikan: writing – original draft preparation, validation, writing – reviewing and editing, Ehiaghe Agbovhimen Elimian: writing – original draft preparation, validation, writing – reviewing and editing, Simiso Dube: writing – original draft preparation, validation, writing – reviewing and editing, Mathew Muzi: writing – original draft preparation, validation, writing – reviewing and editing, Mustapha Omenesa Idris: writing – original draft preparation, validation, writing – reviewing and editing, project administration.

## Conflicts of interest

The authors do not have any financial or non-financial conflict of interest to declare.

## Data Availability

Data sharing is not applicable to this article as no datasets were generated or analyzed during the current study.
